# More than just smiling: how positive emotions are expressed on the face

**DOI:** 10.1098/rstb.2025.0133

**Published:** 2026-09-24

**Authors:** Kunalan Manokara, Irene Teulings, YongQi Cong, Roza Kamiloğlu, Agneta H. Fischer, Disa A. Sauter

**Affiliations:** ^1^ Social Science Research Institute, Duke University, Durham, North Carolina, USA; ^2^ Department of Psychology, University of Oslo, Oslo, Norway; ^3^ Department of Social Psychology, University of Amsterdam, Amsterdam, The Netherlands; ^4^ Faculty of Behavioural and Movement Sciences; ^5^ Department of Experimental Psychology, University of Oxford, Oxford, UK

**Keywords:** positive emotions, spontaneous expressions, facial behaviours, network analysis

## Abstract

To advance the science of positive emotional expressions, we test how 22 positive emotions are spontaneously displayed on the face, using the participant-negotiated episodic recall method. Respondents (*n* = 163) narrated emotional events from their lives, and we extracted facial behaviours that reflected peak expressivity (35 410 facial actions). Network models were applied to probe whether specific positive emotions would be conditionally associated with particular facial behaviours. Our results point to three sets of positive emotions: five that do not seem to have specific facial actions related to them (e.g. admiration), nine whose expressions do not significantly involve smiling (e.g. head turn for interest, raised chin for determination), and eight whose facial expressions include smiling as a unique expressive feature (e.g. positive surprise and elation). Notably, nine of these positive emotions had expressive patterns that were distinct from the other emotions in our set (e.g. raised cheeks and closed eyes for sexual desire), while eight emotions were associated with expressive patterns that were shared in pairs (e.g. raised eyebrows for both hope and inspiration). These results provide a tentative map of what specific positive emotions look like when produced on the face in a naturalistic situation.

This article is part of the theme issue ‘Mechanisms, development, phylogeny and functions of emotional expressions’.

## 1. More than just smiling: how positive emotions are expressed on the face

Feeling good is part of most people's everyday lives, and we often express positive feelings as we experience them: we laugh when amused by a joke, smile when we see a familiar face in a crowd, and sigh at being relieved that a difficult experience is coming to an end. Our emotional behaviours often occur in a spontaneous manner: either in the moment [1], or when talking about it afterwards [2,3]. While a substantial body of work has detailed how specific emotions are expressed, the vast majority of these expressions were posed [4,5]. Critics have pointed out that empirical findings based on emotional displays may not generalize to expressions in real life [6]. Charting emotional expressions based on naturalistic behaviours therefore remains a critical next step for advancing our understanding of how we express ourselves when we feel good (e.g. [7]).

In the current research, we examine how different positive emotions are spontaneously expressed. Given the importance of facial cues in communicating emotional states [8,9], we focus on how a broad range of positive emotions are expressed on the face. We hence employ established methods to elicit [10] and delineate [11] the facial expressions of 22 positive emotions. We then subject these expressions to network analyses, in order to examine whether particular facial actions are conditionally associated with specific positive emotions.

## 2. What we know about positive emotions and their expressions

We operationalize positive emotions as states that people experience to be pleasant (see [12]). In recent years, psychologists have made steady progress in empirically establishing how specific positive emotions like awe [13], gratitude [14] and admiration [15] are subjectively experienced [16], expressed when cued [4], perceived by others [17] and relate to various action tendencies [18]. However, positive emotions have often been studied in isolation rather than comparatively. Examining positive affective states in tandem—rather than in silos—is an essential next step towards building a more comprehensive and nuanced science of emotions. In this paper, we focus on the expressive component of positive emotions, which have received relatively less attention from empirical researchers [19]. Specifically, we examine facial behaviours, given their importance in expressing emotion [20].

Although it was initially proposed that all positive emotions involve Duchenne smiles (i.e. smiling with crow's feet around the eyes: [21]), recent research points to a broader expressive palette (for a discussion of the literature on smiles, see [22,23]). There is growing acknowledgement in affective science that some positive emotions are expressed with specific distinguishable nonverbal behaviours [1,24,25], including facial expressions (e.g. smiling for elation: [4], raised eyebrows for awe: [26]) that could be subtle (e.g. greater frequency of smiling for pride as compared to interest pleasure: [27]). It is also worth noting that for some positive emotions, unique expressive facial configurations have not been found even though empirical attempts have been made (e.g. gratitude: [28]). In addition, there exists a range of positive emotions (e.g. hope, moral elevation) that have been predominantly examined from the perspective of experience-related outcomes (e.g. intergroup helping: [29]; prosocial behaviours: [30]). Researchers theorize that the social sharing of these emotions could amplify the positive societal outcomes associated with them (see [31]), hence making the study of their expressive cues important. However, systematic attempts to empirically chart such behaviours are scarce (for a discussion see [32]).

## 3. What remains to be known about positive emotional expressions

Two major caveats limit our current understanding of how positive emotions are expressed. The first relates to an incongruence between theories on emotional expressions and the predominant methods. Early theories of human emotion [33,34] focused on how people ‘produce’ facial and bodily expressions. However, most empirical research on emotional expressions uses ‘perception’ paradigms, which test whether people can identify others’ emotional expressions accurately (see [19]). Using results from one angle of inquiry (e.g. whether emotions are recognizable) to draw inferences about another (e.g. how emotions are produced) can be conceptually and empirically problematic. Emotional expressions can contain both signals (which have been selected for through evolution to serve communicative purposes: [35–37]), and signs (a by-product of behaviour serving another primary function: [33,38,39]). Hence, an emotional expression could involve behaviours that may not have been selected for—in evolutionary terms—as social signals. If perceptual judgments are the only means through which scientists investigate emotional expressions, then some behaviours that are inherent parts of an expression could remain undocumented because human judges do not detect those cues.

Nevertheless, some of these behaviours could be consequential for the expresser, most notably by serving an intrapersonal purpose (see [40]). For example, sighing when relieved serves a resetting function (i.e. helps the expresser's breathing transition from a state of stress back to baseline: [41]), while emotional tears (such as when feeling moved: [42]) are purported to be an emotion regulation mechanism (i.e. cathartic release from being overwhelmed: see [43]). To build a more comprehensive and accurate understanding of how positive emotions are expressed, conducting production studies is vital (for a deeper discussion on conceptual distinctions between production and perception, see [44]).

Secondly, in the small number of production studies that have been conducted, the emotional expressions are typically posed (see [1]). Posed displays of an emotion have been argued to reflect stereotypical understandings of how an emotion is expressed in a given culture rather than actual expressions (see [45]). Critics claim that such expressions may thus reflect peoples’ stereotypical understanding of how an emotion is expressed, rather than actual expressions per se. Posed expression paradigms have also been criticized for using standardized emotion elicitors across participants [46]. Yet, what causes interest differs between individuals [47], and what constitutes a sexually desirable target varies substantially between people [48]. Using scripted episodes therefore fails to capture the richness of situations that can elicit an emotion, which may inflate the consistency in how that emotion is found to be expressed.

Recent research is starting to address some aspects of these criticisms, including investigating spontaneous displays in perception studies [49]. In fact, perception paradigms have advanced considerably and yielded fresh insights. A range of positive emotions can be identified with accuracy from facial expressions, even when people classify naturalistic facial expressions (see [50]), and considerable work has compared accuracy rates between posed and spontaneous expressions of smiling (see [51]). In summary, there is interest in understanding how positive emotions are expressed spontaneously, yet work to date has primarily been conducted through a perception lens (i.e. testing whether observers can identify an emotion); there remains a relative absence of research investigating the expressions of positive emotions themselves. This imbalance is understandable; there are significant feasibility barriers for conducting production studies, given that data collection and pre-processing are time-intensive and costly, particularly with well-powered sample sizes. However, such effortful work is necessary to advance our understanding of what specific positive emotions look like when produced spontaneously on the face.

## 4. The current research

In the present research, we aimed to answer the question of how a broad range of 22 positive emotions are expressed on the face. To elicit each of these emotions in an unscripted manner, the participant-negotiated episodic recall method was used ([10], also see [49]). In this method, people are asked to recount specific situations from their past (e.g. a time when they experienced a particular emotion) and are video recorded while narrating their stories. To taxonomize facial behaviours relating to each emotion, we applied the Facial Action Coding System (FACS: [52]) to the videos. In order to examine the degree to which specific facial behaviours (e.g. smiling) would be conditionally associated with each positive emotion (e.g. gratitude), we conducted network analyses with these data. In essence, our work aims to provide an initial map of what specific positive emotions may look like when spontaneously shown on the face.

## 5. Methods

### (a) Statement on ethics, transparency and openness

Ethical approval was obtained from the Research Ethics Committee at the University of Amsterdam: 2018-SP-9223. All analyses were performed in R version 4.3.2 [53]. We report how we determined sample size, all data exclusions, and study materials. Anonymized data and code are available at: https://osf.io/djfnw/?view_only%3D44fff51a2c664f17bb85e9804be2ac90.

### (b) Participants

*A priori* power analyses were difficult to conduct for two reasons: a lack of readily available effect sizes for spontaneous emotional expressions, and the complexity of the statistical models given our use of network analyses.^1^ We hence took direction from sample sizes used in the literature and additionally conducted sensitivity analyses post data collection. Studies on posed emotional expressions have typically recruited between 50 and 80 respondents per population (e.g. [4,28]). Given that spontaneous expressions may be less prototypical and therefore less differentiable ([27] we aimed for a sample size that was double that of previous studies (i.e. target *n* = 160). In total, we recruited 163 native, Dutch-speaking (as first language) participants between 18 and 34 years old (*M*_age_ = 21.3, 126 women, 37 men), who had completed high school and did not report any mental health conditions or history of voice, speech or language disorders (see the supplementary material S4 for further demographics). Participants were compensated with €40 each. Sensitivity analyses demonstrated that our sample was sufficiently powered at 80% for detecting small to medium effects in frequency distribution analyses (*w* = 0.26). Data collection took place from 2018 to 2020.

## 6. Materials

### (a) Selecting positive emotions

To determine which positive emotions to examine, we adopted a four-step process: we (a research team consisting of five affective scientists from multiple cultural backgrounds) started with a generative process of listing positive emotion terms. Next, we collated a list of emotions that have been studied empirically or theorized about. We then culled emotions for which there was no data on experience, expressions, or outcomes (e.g. feeling ‘strong’ or ‘connected’: also see [54]). Finally, we excluded pleasant feelings that last longer than a typical emotion would (e.g. being calm or peaceful). We also excluded emotions that are beneficial in terms of intrapersonal outcomes, yet do not typically feel good (e.g. compassion: [55]). This process led to a final list of 22 positive emotions: admiration, amae, amusement, awe, being moved, determination, elation, excitement, feeling respected, gratitude, hope, interest, inspiration, moral elevation, positive surprise, pride, relief, schadenfreude, sensory pleasure, sexual desire, tenderness and triumph. While comprehensive, this set of positive emotions is not meant to be exhaustive.

### (b) Eliciting positive emotions

Choosing a paradigm to elicit emotional expressions requires balancing between experimental control (e.g. posed expressions) and ecological validity (e.g. naturalistic expressions: for a detailed explanation, see the supplementary material, S1). Here, we opted for what we believe to be a middle ground: the participant negotiated episodic recall method [10]. In this method, people are asked to recount specific situations from their past (e.g. a time when they experienced a particular emotion), which is psychologically involving as it requires the recollection and sharing of personal emotional experiences [56]. Participants are video recorded in the laboratory while freely narrating their stories to the camera; hence, experimental control and data quality are high, and emotional experiences are known to be evoked effectively [57]. As this method uses participant-selected emotion elicitors drawn from their personal experiences—rather than standardized stimuli—it captures the diversity of situations that can trigger a specific emotion.

### (c) Measuring emotional experience

We collected self-report measures of emotional experience to evaluate paradigm validity.^2^ For each of the 22 positive emotions, we measured how intense the experience of the target emotion was (1 = *not at all*, 7 = *very much*), first during recall, and then at the time of the actual event. In addition, for each positive emotion, we also measured perceived social engagement, valence, and physiological arousal (see the supplementary material, S5 for full materials).

### (d) Measuring emotional expressions

While there are a variety of techniques to describe facial behaviours, the gold standard for facial expression analysis is the FACS [59]. It is an anatomically based comprehensive coding scheme that taxonomizes the movements of facial muscles, referred to as action units (AUs). In FACS, each facial behaviour (e.g. lip corner puller) is assigned a code (e.g. AU12). While initially created for manual use by human coders, in recent years, FACS is increasingly used to develop automated methods based on deep learning algorithms. Such machine-based techniques can be useful for analysing large datasets, which are typical in studies of spontaneous expressions, given that such data are typically represented with video segments (constituting multiple frames).

### (e) Analysing facial expressions

Statistical techniques for analysing facial expressions have developed in recent years, with one advance involving the application of network models to AUs [60]. Network models test for associations between facial behaviours and emotions, based on conditional probabilities, that is, the likelihood of an AU being activated for a given emotion while considering its occurrence during other emotions as well. Rather than using a pre-selected chance threshold for all facial behaviours (i.e. classical frequency analyses with non-parametric tests), network models instead operate based on adjusted thresholds that are specific to each AU, thereby controlling for occurrence across the entire dataset. Some facial behaviours may simply occur more or less often in general, regardless of emotion (e.g. smiling may occur frequently across all positive emotions). Network models thus probe whether AUs are associated with a given emotion while controlling for differences in base rates. A central advantage of this approach is that it can provide degrees of specificity; network models help metricize how differentiable each emotion is when contrasted against others. In summary, network models illustrate the extent to which facial expressions are relatively more associated with specific emotions as compared to their occurrence across the data.

## 7. Procedure

### (a) Pre-recording preparation

Participants signed up for an in-person laboratory study that would require them to narrate past experiences involving each of 22 positive emotions in sequence, while being video recorded. Three days prior to their laboratory appointment, they were e-mailed the emotion terms and definitions (see the supplementary material, S2) and asked to note down specific life events during which they had experienced each of the 22 target emotions.

### (b) Laboratory set-up and equipment

Participants were alone when narrating their experiences; the experimenter was in an adjacent room. Participants’ privacy was considered important given display rules for positive emotional expressions [61]. Participants were seated in front of a camcorder (Canon LEGRIA mini-X) with full high definition recording capabilities. A white screen backdrop was installed, and the walls were lined with soundproofing sheet, to minimize distractions and to control light conditions. Adjacent to the recording chair, we also placed a computer (and chair), which participants used to answer self-report items (see the supplementary material S5 for complete details).

### (c) Recording procedure

Participants were video recorded during the entire study session, which was conducted in their mother tongue (Dutch). They were first seated in the computer chair, where they were cued with one of the 22 positive emotions (presented in random order via the laboratory computer). The experimenter verbally checked that the participant understood the meaning of the cued emotion by asking for a definition in the participant's own words. Thereafter, the experimenter left the room, and the participant moved to the recording chair to narrate their life experience of the cued emotion (towards the camcorder). Upon finishing their narration, participants moved back to the computer chair to answer questions about their emotional experience. The next emotion was then cued, and this process was repeated 22 times. To mitigate against fatigue, at the halfway point of the experiment (i.e. after 11 emotions), we provided a 15 minute caffeine break. In general, it took participants approximately 150 minutes to complete the full study.

### (d) Data preparation

At the end of the experiment, after another short 5 minute break, participants watched their recordings. They were asked to self-select a video segment (between 2 and 5 seconds long) for each positive emotion, where they believed they expressed that emotion most intensely (i.e. the expression apex: [52]). This self-selection approach aligns with the spirit of the participant-negotiated recall method [10,56]. If a participant was unable to recall an emotion, or did not wish to share an experience of a certain emotion, we excluded it. The final dataset consisted of 3541 video segments.

We applied FACS [52] to the participant-selected video segments. FACS describes facial expressions in terms of 58 AUs that index visible facial muscular activity and can be applied to data using either human coders (e.g. manual coding) or by employing software (e.g. automated coding). For our research, we used FaceReader's automated facial expression analysis software (Version 9, Noldus), which scores the occurrence of 25 AUs. See the supplementary material S3 for further details on the FACS and automated coding.

Given that we obtained approximately 3541 video segments, and each segment was coded in terms of 25 facial actions, our final dataset consisted of 88 525 binary data points (i.e. each AU either being present or absent in each emotion expression). To evaluate the validity of the automated coding in FaceReader, we additionally applied manual coding to a subset of the participant-selected video segments. A certified FACS coder with 5 years of experience—author K.M.—manually coded 264 video segments (7.5% of our dataset). These segments were selected in a quasi-random manner, such that participants and emotions were fairly represented. The coder was unaware of which specific emotion was being displayed in each video, and the coding took approximately 200 hours to complete.

## 8. Results

All statistical analyses were conducted using R version 4.3.2 [53].

### (a) Paradigm validity

We first examined if our paradigm successfully elicited the intended positive emotions. For all 22 positive emotions, self-reported intensity of the target emotion during the episode was recorded to be significantly above the scale midpoint (range from 1 to 7; *t*s > 11.41, *p*s < 0.001, Cohen's *d*s > 0.92). This indicates that participants’ selected episodes induced the emotions as intended (all *M*s > 5.27). For intensity during recall, 19 emotions were reported to be experienced at levels that were significantly above midpoint as well (*t*s > 4.25, *p*s < 0.001, Cohen's *d*s > 0.33), with three exceptions: sexual desire (*M* = 4.01, s.d*.* = 1.48), schadenfreude (*M* = 4.31, s.d. = 1.53), and sensory pleasure (*M* = 4.35, s.d. = 1.56). Hence, even during recall, most of the emotion episodes elicited the intended emotions fairly intensely. Taken together, these results provide confidence in the efficacy of the participant-negotiated recall method to elicit the target emotions. For further details on self-reported emotion experience, see the supplementary material, S5.

### (b) Convergent validity between FaceReader and manual coding

Researchers have raised validity concerns regarding automated facial coding; algorithms are known to miss minute facial movements, and because of hypersensitivity to light, are prone to mistake shadows on the face for muscle movement and hence over-code [62,63]. We thus began by evaluating convergent validity between FaceReader output and our manual FACS codes. All analyses were conducted at the AU level, and we used established techniques to examine convergence (see [64]): We first calculated ratios of recall (e.g. manual codes that are detected by FaceReader) and precision (e.g. correct classification by FaceReader when a manual code is indicated as present by FACS coding). Next, we assessed *F*1 indices, which are calculated as two times the product of precision and recall, divided by the sum of precision and recall, to arrive at the so-called harmonic mean of recall and precision. We provide an overview of comparisons between manual and machine-based FACS coding in the supplementary material, S3. To achieve balance between conservative and inclusive criteria, we applied a validity threshold of *F*1 ≥ 0.65.

Of the 25 AUs that FaceReader coded, acceptable convergent validity with manual FACS codes was obtained for 10 facial behaviours (35 410 binary data points). The remaining 15 AUs were hence excluded from all subsequent analyses. We applied this approach to retain only valid data points, while minimizing false positives and false negatives (further details in the supplementary material, S3, table S1). For the 10 AUs that passed our threshold, the model is performing reasonably well: it correctly identifies most of the relevant cases (high recall) while keeping the number of false positives relatively low (good precision). These 10 AUs are: inner brow raise (AU1), outer brow raise (AU2), cheek raise (AU6), lid tightener (AU7), lip corner puller (AU12), chin raise (AU17), lips part (AU25), jaw drop (AU26), eye closure (AU43), and head turn right (AU52).

### (c) Applying network models to emotional expressions

We sought to examine whether specific AUs would be conditionally associated with specific emotions (i.e. that a given AU would occur more frequently for a hypothesized emotion than for other emotions), thus taking into account overall frequency rates for each AU across the 22 positive emotions. The network modelling approach (NetFACS package: [60]) demonstrates connections between AUs and conditions based on the conditional probabilities of AUs being activated in each condition (in our case, emotion). These conditional probabilities are established by testing the observed probability of an AU (how often the AU is observed) against its expected probability (how often the AU would be expected to be observed), which is a bootstrapped reflection of the overall occurrence of that AU across emotions. The bootstrap was performed on individual level, so that the null distribution accounted for inter-individual differences and autocorrelation across videos.

In other words, the network models identify which facial actions are more likely to be active for specific emotion expressions, accounting for how often those facial actions occur in general across the dataset (for raw occurrence rates, see the supplementary material, S5). Edges in network models are commonly interpreted as direct or unique associations between variables, reflecting relationships that persist after conditioning on all other nodes [65,66]. Building on this interpretation, we use the term conditional associations to emphasize that they reflect statistically isolated relationships rather than raw co-occurrence. Aligned with the published literature [67,68], we interpret significant conditional associations to reflect unique relationships (i.e. after controlling for the other variables in our dataset). The network modelling approach hence controls for the imbalance of overall frequencies, and thereby tests whether particular AUs are conditionally associated with specific emotions. This is especially important for spontaneous expressions, given the inflated likelihood of participants’ lips parting (AU25) given they are talking to the camera.^3^

To map out indices per emotion, we first conducted 22 network models, taking into account the overall AU occurrence rates across our full dataset (see figure 1). These network graphs display the results of our models, depicting the statistical relationships (occurrence probabilities) between emotions and AUs. AUs that were significantly associated with each emotion (*p* < 0.05) are highlighted via green circles. Edges (visualized as connecting lines) reflect partial associations between different AUs for a specific emotion and are assigned only if the conditional probability of both AUs occurring for that given emotion expression is significant (*p* < 0.05), meaning that the observed probability is higher than the expected probability in 950 out of 1000 bootstraps of the null condition.

**Figure 1.**
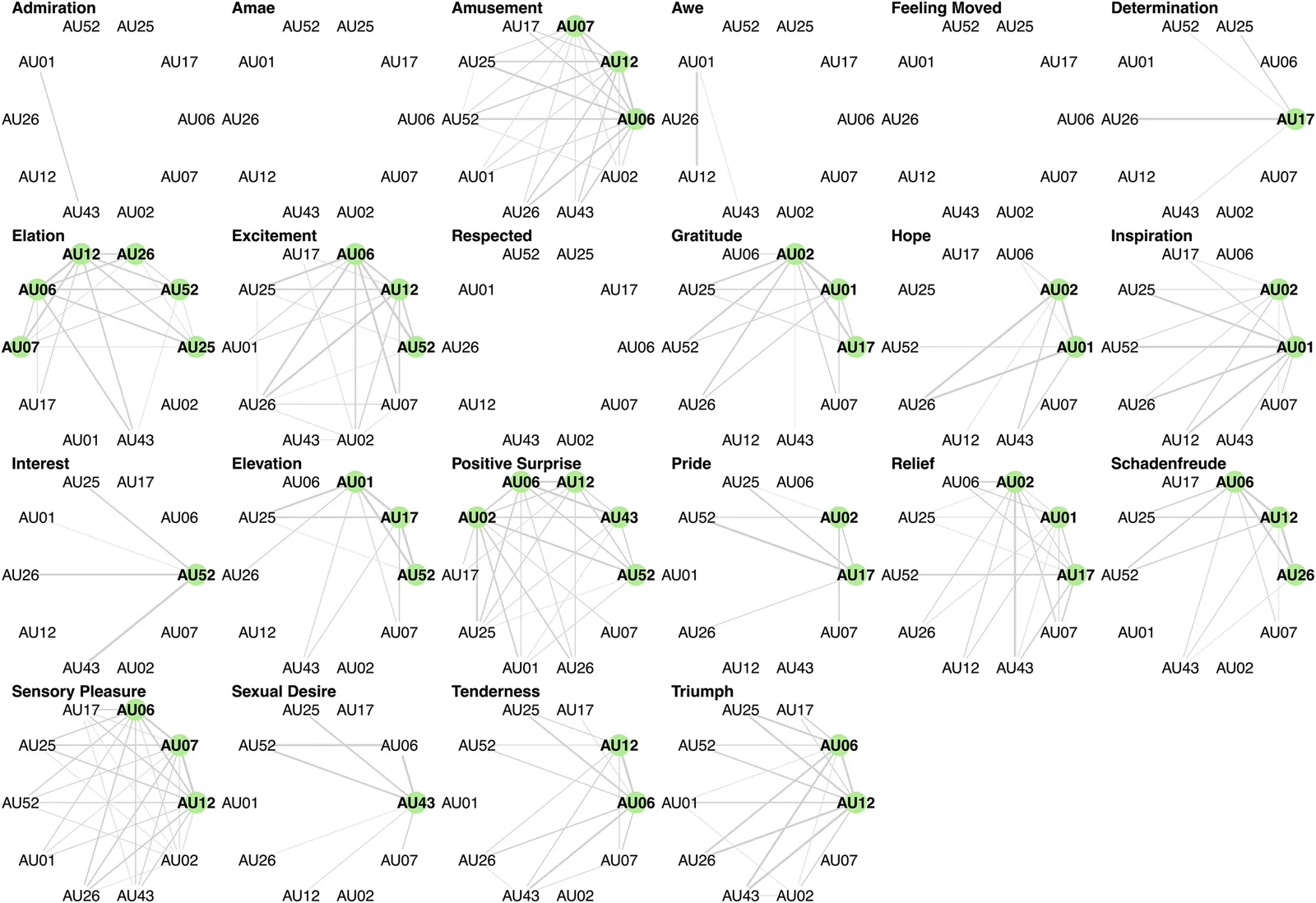
Network models for each of the 22 positive emotions. Note*:* significant AUs for each emotion are highlighted with green circles. The number of edges reflects how connected AUs are with one another, with thicker edges signalling a higher likelihood of co-occurrence of two AUs.

Two key findings come to light when visually inspecting the network models in figure 1. First, for five of the positive emotions in our set (admiration, amae, awe, being moved, and feeling respected), we did not find any significant associations with the facial behaviours examined in this study (i.e. absence of green circles). The remaining 17 positive emotions were found to be conditionally associated with at least one AU (i.e. presence of green circles; see table 1 for summary of results).

**Table 1.** Density indices and facial behaviours conditionally associated with each positive emotion. (Note: density indices (italicized) denote the ratio of the number of observed edges divided by the number of possible edges; networks with high density have connections between most nodes, suggesting that facial expressions in this condition either comprise more AUs than in other conditions, or that AUs co-occur in more diverse combinations. Occ refers to occurrence frequencies and Δ refers to probability increase rates (e.g. for interest, head turning occurs in 96% of our sample, and is 6% more likely to occur for interest than across emotions).)

emotion	complexity of expression (density index)	unique facial behaviours (action unit; occurrence rate; probability increase)
amae	*0.09*	—
being moved	*0.09*	—
feeling respected	*0.20*	—
admiration	*0.31*	—
awe	*0.53*	—
interest	*0.31*	head turn right (AU52; occ = 96%; Δ = 6%)
determination	*0.40*	chin raise (AU17; occ = 74%; Δ = 4%)
pride	*0.40*	outer brow raise (AU2; occ = 41%; Δ = 7%);
		chin raise (AU17; occ = 75%; Δ = 6%)
sexual desire	*0.44*	cheek raise (AU6; occ = 73%; Δ = 4%);
		eyes closed (AU43; occ = 80%; Δ = 5%)
moral elevation	*0.31*	inner brow raise (AU1; occ = 58%; Δ = 14%);
		chin raise (AU17; occ = 79%; Δ = 11%);
		head turn right (AU52; occ = 92%; Δ = 2%)
hope	*0.58*	inner brow raise (AU1; occ = 55%; Δ = 7%);
		outer brow raise (AU2; occ = 42%; Δ = 9%)
inspiration	*0.47*	inner brow raise (AU1; occ = 61%; Δ = 20%);
		outer brow raise (AU2; occ = 43%; Δ = 13%)
gratitude	*0.62*	inner brow raise (AU1; occ = 56%; Δ = 10%);
		outer brow raise (AU2; occ = 45%; Δ = 18%);
		chin raise (AU17; occ = 74%; Δ = 5%)
relief	*0.82*	inner brow raise (AU1; occ = 55%; Δ = 7%);
		outer brow raise (AU2; occ = 42%; Δ = 9%);
		chin raise (AU17; occ = 75%; Δ = 6%)
excitement	*0.64*	cheek raise (AU6; occ = 79%; Δ = 13%);
		lip corner puller (AU12; occ = 81%; Δ = 1%);
		head turn right (AU52; occ = 93%; Δ = 2%)
schadenfreude	*0.38*	cheek raise (AU6; occ = 79%; Δ = 12%);
		lip corner puller (AU12; occ = 82%; Δ = 11%);
		jaw drop (AU26; occ = 92%; Δ = 3%)
positive surprise	*0.82*	outer brow raise (AU2; occ = 47%; Δ = 23%);
		cheek raise (AU6; occ = 78%; Δ = 11%);
		eyes closed (AU43; occ = 80%; Δ = 5%);
		lip corner puller (AU12; occ = 80%; Δ = 8%);
		head turn right (AU52; occ = 93%; Δ = 3%)
elation	*0.71*	cheek raise (AU6; occ = 79%; Δ = 13%);
		lid tightener (AU7; occ = 75%; Δ = 5%);
		lip corner puller (AU12; occ = 83%; Δ = 13%);
		lips part (AU25; occ = 99%; Δ = 2%);
		jaw drop (AU26; occ = 91%; Δ = 3%);
		head turn right (AU52; occ = 93%; Δ = 3%)
tenderness	*0.44*	cheek rais**e** (AU6; occ = 81%; Δ = 15%);
		lip corner puller (AU12; occ = 80%; Δ = 9%)
triumph	*0.67*	cheek raise (AU6; occ = 77%; Δ = 10%);
		lip corner puller (AU12; occ = 82%; Δ = 11%)
amusement	*0.87*	cheek raise (AU6; occ = 90%; Δ = 28%);
		lid tightener (AU7; occ = 76%; Δ = 8%);
		lip corner puller (AU12; occ = 89%; Δ = 21%);
sensory pleasure	*0.98*	cheek raise (AU6; occ = 80%; Δ = 14%);
		lid tightener (AU7; occ = 80%; Δ = 13%);
		lip corner puller (AU12; occ = 83%; Δ = 14%);

Second, while some positive emotions were observed to have a large number of edges connecting multiple facial behaviours (e.g. amusement), others were instead found to have a smaller number of edges connecting AUs (e.g. determination). The extent to which edges connect separate AU nodes denotes the complexity of an emotional expression—metricized with a density index, which is based on the ratio of actually existing edges in a network relative to all possible edges [69]. When visualized, a high-density network would have multiple edges running across nodes (e.g. sensory pleasure), while a low-density network would only have a few edges that connect nodes (e.g. pride). These differences in density can help reveal which emotions have more complex facial patterns that involve multiple AUs (e.g. positive surprise) versus simpler ones (e.g. interest). In table 1, we list these density indices for each positive emotion.

Table 1 summarizes the results from the network analyses.^4^ For each positive emotion, we report which facial movements were found to conditionally occur, how frequently they occurred, how much more likely their occurrence was deemed to be when compared to the overall dataset (e.g. for determination, chin raising occurs in 74% of our sample, and is 4% more likely to happen for determination than in general across the sampled positive emotions), and density indices. Of the 17 positive emotions that were conditionally associated with facial behaviours, nine emotions did not include smiling as a uniquely associated expressive feature. For example, determination was conditionally expressed with raised chins, while hope and inspiration were both associated with raised eyebrows.

The remaining eight positive emotions were all found to contain smiling as a uniquely associated expressive feature. For example, amusement and sensory pleasure both involved Duchenne smiles, while excitement was characterized by smiling and horizontal head movements. Taken together, our findings point to the 22 positive emotions making up three broad sets (see table 1): five that do not seem to have specific facial actions related to them, nine whose expressions are not conditionally associated with smiling (of which five have expressive patterns that upon inspection look different from the other emotions), and eight whose facial expressions include smiling (of which four have facial patterns that are different from others).

To provide a visual summary of which facial actions are conditionally related to each positive emotion, we graphed a network model to visualize the full set of statistically significant AU-emotion links (figure 2). This network model characterizes the occurrence probability of AUs in emotions; only AUs that were significantly more likely than expected (based on the null condition, at *p* < 0.05) show links to that emotion. The edge weights represent the conditional probability of the AU occurring in an emotion (e.g. the probability of AU17 (chin raiser) being active in a pride expression is 0.75).

**Figure 2.**
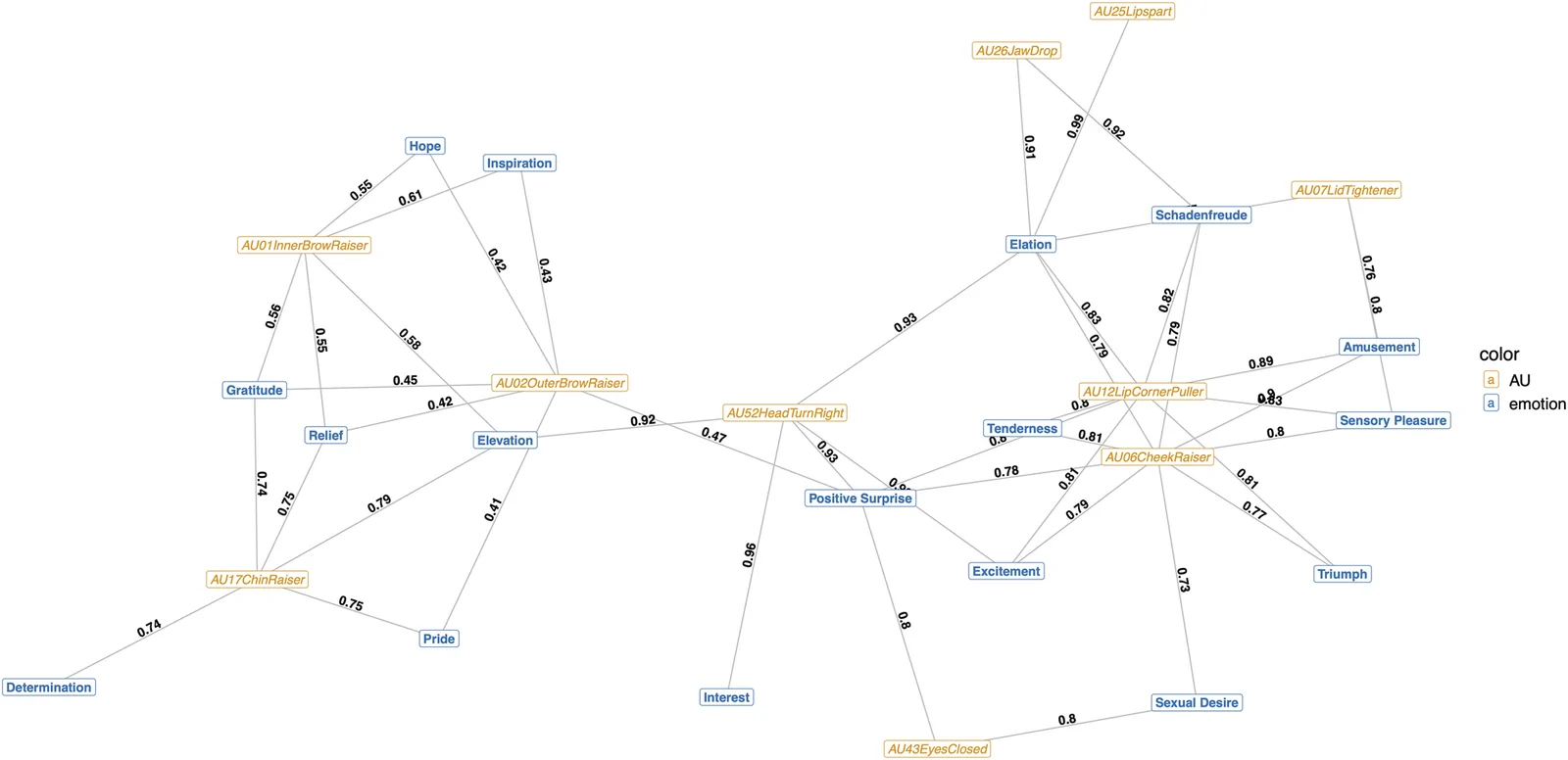
Full network model of statistically significant emotion expressions. Note: this network model visualizes significant connections between AUs and emotions (emotions for which no AUs were significantly related do not appear in the graph). Bolded numbers denote the occurrence of each connection (i.e. the conditional probability to observe an AU given an emotion). For example, if pride is the emotion that is being expressed, AU17 (chin raiser) occurs in 75% of the expressions.

## 9. Discussion

In this study, we sought to examine how specific positive emotions are expressed on the face. To this end, we used established methods to elicit [56] and taxonomize [59] the facial expressions of 22 positive emotions. We then conducted network analyses to probe whether each emotion is associated with a particular set of facial behaviours. This analysis revealed three sets of positive emotions, the first of which encompassed five emotions that did not map to specific facial behaviours (e.g. admiration). A second set of nine emotions had expressive patterns that did not uniquely entail smiling. Five of these emotions had expressive patterns that were distinct from the other emotions in our data (e.g. horizontal head movement for interest), while four of these emotions shared expressive patterns in pairs (e.g. raised eyebrows and chins for both gratitude and relief), with each pair demonstrating distinctiveness from other emotions. A third set of eight emotions were uniquely expressed with facial actions that included smiling, four of which had unique expressive patterns (e.g. smile, wrinkle around the eyes, and jaw drop for schadenfreude). The remaining four emotions shared expressive patterns in pairs (e.g. smiling and wrinkling around the eyes for both tenderness and triumph), with each pair again demonstrating distinctiveness. Taken together, our findings point to 13 patterns of facial expressions that were uniquely related to particular (pairs of) positive emotions, of which seven expressive patterns did not include smiling. Not all pleasant feelings look the same when they are expressed, and relatedly, not all states that make people feel good are necessarily shown with a smile.

### (a) Differential expressions across (sets of) positive emotions

For five emotions (admiration, amae, awe, being moved, and feeling respected), specific facial configurations in relation to each of them did not emerge from our network models. We note that a unique emotional expression for awe has yet to be found [4], while the remaining emotions have predominantly been examined from the perspective of subjective experience (admiration: [15]; being moved: [43]; amae: [70]). There are at least three explanations for our findings. First, the facial behaviours represented in our data do not include the complete repertoire of facial movements that could possibly occur on a human face. It may be that some facial actions which did not pass convergent validity thresholds in our data could contain important information for specific emotional expressions. Second, some positive emotions are known to have characteristic non-facial behavioural components, that are not typically coded with FACS. These include human tears, which are a key feature of feeling moved [42], and chest expansion with arms akimbo when feeling authentic pride [71]. A third explanation for our findings could relate to emotion display rules [61], which are particularly strong for emotions that may signal vulnerability (e.g. feeling moved: [72], and possibly amae too, given it involves submission to a benevolent authority: [70]), and relatedly for intense emotional expressions (see [73]). Although we ensured privacy in the laboratory, being aware of a video recording could have motivated participants to apply display rules and hence down-regulate their expressions of some emotions. In summary, our results for this final set of emotions merits further inquiry and should not be taken to demonstrate that differentiable expressions for these emotions do not exist, although they are compatible with that possibility.

A second set of nine emotions were found to have facial expressivity patterns that did not uniquely include smiling. Interest expressions specifically involved head movement to the side (potentially indicative of a need to break eye contact for the sake of effortful thinking: [47]). Determination (and pride) were expressed with raised chins (in addition to raised outer eyebrows for pride), perhaps reflecting the recollection of socially valued traits such as competence (see [71]). Hope was conditionally expressed with raised eyebrows (perhaps indicative of expressions that denote anticipation and thinking about the future, see [29]), and inspiration was characterized by the same pattern (aligned with theorizing about inspiration being both epistemological and future-oriented, colloquially termed the ‘Eureka’ moment: see [74]). Sexual desire was characterized by eye closure, which is a typical behaviour for savoring positive emotions (see [19]), and possibly reflects enjoyment through intentionally recalling the pleasurable stimulus. Together, these emotions suggest that not all that feels good seems to be uniquely expressed with a smile, adding nuance to the suggestion that AU12 could be a common signal for all positive emotions (also see [27]).

A final set of eight emotions were expressed with facial actions that included smiling as a unique expressive cue. There are at least two common elements that feature across these eight emotions (e.g. excitement, positive surprise, amusement). First, they could be classified as high-arousal positive states (see [75,76]). Perhaps when a pleasant experience is physiologically arousing, smiling becomes characteristic of its expression, although further empirical work is needed to establish this possibility. Second, the eliciting stimulus for each of these emotions is typically present-oriented in time scale: people feel triumphant at the point of victory [77], excited when they are delivered good news [78], amused when they hear a joke [79], sensory pleasure when stimulated [80], and schadenfreude at observing the deserved misfortune of another [81]. It is hence possible that a smile is a characteristic feature of (high arousal) positive states that are experienced in-the-moment, as opposed to positive emotions that require explicit recall from the past (e.g. gratitude: [14], pride: [71]) or even thinking about the future (e.g. hope: [82]; inspiration: [83]).

### (b) Limitations and future directions

By applying a network modelling approach to expressions data, we were able to pinpoint how particular facial behaviours (e.g. cheek raising) express specific positive emotions (e.g. 28% more likely for amusement than across the set of 22 positive emotions). Although informative (and statistically significant), the effects should be classified as small, and thus should be interpreted as such. As acknowledged by statisticians, a key limitation of the network modelling approach is that results are heavily dependent on the included set of variables [84]; the conclusions we draw from our findings hence relate specifically to contrasts between these 22 positive emotions. In fact, when drawing from examples where more prototypical emotional expressions are compared against each other (e.g. anger in a dataset of seven basic emotions), specific AUs can be multifold more likely to occur per emotion (e.g. frowning is 200% more likely when posing anger than other basic emotions: [60]). Indeed, if positive and negative emotions are contrasted against each other, larger effect sizes would probably emerge. Given that our dataset consisted of a large range of positive emotions that probably have overlap in terms of experience [58] and facial movements (see also [27]), less specificity is to be expected.

While our choice of method to elicit emotion attempts to balance between experimental control and ecological validity (elaborated in the supplementary material, S1), three limitations should be noted. First, we acknowledge cross-emotion differences in participants’ recall intensity; the experience of schadenfreude, sexual desire, and sensory pleasure may be strongly tied to situational elicitors, which could explain why intensity was reported to be weaker during recall. For these emotions, facial expressions in our data are possibly less representative of contemporaneous experience. Second, we recognize that in addition to the target emotion, participants could have been experiencing other emotions when narrating stories (e.g. mixed emotions: [85]). However, it was simply unfeasible to include multi-item experience measures in each condition, given the length of the study. Third, as speech is an inherent part of spontaneous expressions, our results for lip parting (AU25) encompass both speech-related and non-speech-related actions; we are unable to distinguish between the two solely with the present data.

In the current research, we employed the apex approach (i.e. examining the most intense point of each expression) to determine which part of a video segment to analyse, which is a commonly used FACS coding approach. Yet doing so quashes the variance in expressivity patterns that could occur across timepoints. Given that facial expressions change over the course of an emotional episode (e.g. appraisal related adjustments to the display of triumph: [77]), specificity for positive emotional expressions may be greater if temporal dynamics were accounted for. For a richer understanding of how emotional expressions spontaneously unfurl, frame-by-frame analyses will be needed. On the point of dynamic expressions, we offer a word of caution on interpreting the co-occurrence of facial actions. As our network models were based on the presence of AUs over 2-5 second-long videos (rather than still images), the links between AUs cannot be interpreted as co-occurrence of these AUs in real time; facial behaviours might have occurred sequentially.

Our findings are also limited by the constraints of the algorithm we depended on for automatic facial movement analysis. While FACS taxonomizes more than 50 facial behaviours, only a selection (25 AUs) of these actions is currently available in FaceReader. In the present study, 15 actions were excluded owing to inadequate convergent validity with human FACS coding (thus 10 AUs were included in our final analysis). We of course recognize that only a single human FACS coder was assigned to this dataset. As inter-rater reliability could not be assessed between manual codes, we do not preclude the possibility of the algorithm performing better than credited by the current convergent analysis. Nonetheless, we also note that FACS coding is extremely time consuming (e.g. it takes between 30 and 45 minutes to code a 5 second video segment) and resource intensive (e.g. FACS coders can be expensive to hire given their niche skillset). Given the already laborious investment required for conducting a well-powered in-person production study, we opted for a single human coder. Yet it remains important to acknowledge that we were only able to examine a limited subset of facial movements. We hope that future versions of automated FACS software will demonstrate enhanced validity and greater comprehensiveness in terms of the AUs included, especially because automated methods have steadily increased in classification accuracy with updated versions [52].

### (c) Implications and contributions

To our knowledge, the current research marks the first attempt at trying to answer the question of whether positive emotions—when expressed in a naturalistic setting—are associated with distinct facial behaviours. Some previous work has empirically examined expressivity patterns for specific positive emotions (see the supplementary material, S5), but either using posed expressions recorded in highly controlled settings (e.g. using standardized elicitors: [4]), or by focusing on single emotions through naturalistic observations [86]. Though researchers agree that spontaneous production needs to be studied for a broad range of emotions [1], empirical work in this domain has been limited and mostly used perception paradigms. Our efforts provide new insights into the prevailing understanding of how specific emotions are expressed by examining what an emotion looks like on the face when it is spontaneously produced.

Furthermore, the current research expands our knowledge on some emotional expressions that have received little attention in the literature. There exist multiple positive emotions that have been studied mainly from a subjective experience angle (e.g. moral elevation: [30]), and we here demonstrate that there may be discernable patterns of facial expressions also for such emotions. Our findings hence contribute to theory building for the multiple bodies of work we drew inspiration from (e.g. interpersonal regulation of triumph: [58]; intergroup outcomes of hope: [81]; societal ramifications of awe: [13]), that could be broadly classified under the umbrella of mapping the positive emotion space [16].

## 10. Conclusion

We provide a tentative map of spontaneous facial behaviours for a range of specific positive emotions. Our analyses revealed three sets of positive emotions: some which did not seem to be associated with specific facial actions (e.g. feeling respected), others whose unique expressive patterns did not entail smiling (e.g. interest), and the remaining whose facial expressions were characterized by smiles in addition to other facial behaviours (e.g. amusement). Showing that we feel good thus goes well beyond smiling; the human face is a rich palette through which a variety of positive emotions can be expressed in a wealth of ways.

## Supplementary Material



## Data Availability

Anonymized data and syntax codes are available via the Open Science Framework, at the following link: https://osf.io/djfnw/?view_only%3D44fff51a2c664f17bb85e9804be2ac90. Supplementary material is available online.

## Footnotes

^1^
We acknowledge that simulation-based approaches to *a priori* power analyses have been made available in recent years [52]. However, when we launched data collection in 2018, such tools were not readily available.

^2^
These self-reported items were part of a larger data collection effort, and we thus also measured perceptions of personal involvement (see the supplementary material, S5). A subsection of this data was applied towards a different purpose (i.e. linguistic analyses of narratives) and has since been published (see [58]).

^3^
The R package NetFACS has various functions that extract significant AU-emotion links. Some compare an AUs occurrence in an emotion with its occurrence across the entire dataset (including the emotion itself), while others compare it to its occurrence in all other emotions (excluding the emotion itself). We opt for the first approach owing to its similarity with computing Hu scores (i.e. unbiased hit rates), which is a common statistical technique used in the study of emotional expressions.

^4^
When comparing between computational functions, we note a marginal difference in significance levels for only one particular AU-emotion link. For sexual desire, AU6 was statistically significant when using the overlap_network function (*p* = 0.046), yet was not found to pass the significance threshold when using the netfacs_network function (*p* = 0.051).

## Theme

One contribution of 19 to a theme issue ‘Mechanisms, development, phylogeny and functions of emotional expressions’.
