# Supplementary material for: More than just smiling: how positive emotions are expressed on the face
Source: Philos Trans R Soc Lond B Biol Sci. 2026 Sep 24;381(1959):20250133. doi: 10.1098/rstb.2025.0133 (PMC13644789; doi:10.1098/rstb.2025.0133)
Supplement: Supplementary file 1 [file rstb.2025.0133.zip › rstb.2025.0133/rstb20250133_si_001.docx]

Electronic Supplementary Materials

More than just smiling:

How positive emotions are expressed on the face

Kunalan Manokara^*^

Duke University

Irene Teulings

University of Oslo

YongQi Cong

University of Amsterdam

Roza Kamiloglu

VU Amsterdam

Agneta Fischer

University of Amsterdam

Disa Sauter

University of Amsterdam

**Author Note**

*Correspondence concerning this article should be addressed to Kunalan Manokara, Social Science Research Institute, Duke University, United States of America. E-mail address: manokara.k@duke.edu

**Contents**

Supplement A: Method Details for Eliciting Emotions…………………………..... 3

Supplement B: Emotion Definitions……………………………………………….. 4

Supplement C: Additional Details about the FACS…………………………........... 7

Supplement D: Additional Demographic Information…………………………...... 10

Supplement E: Self-reported emotional experience……………………………….. 11

Supplement F: Inferential and Descriptive Statistics for Action Units……………. 16

**Supplement A: Method Details for Eliciting Emotions**

The most typically used method in the study of emotional expressions is the encoding approach, which uses lab experiments. In the encoding approach, researchers carefully induce specific emotional states, either via audiovisual stimuli (e.g., joyful scenes from a movie: Johnson & Fredrickson, 2005) or preselected narratives (e.g., stories that relate to specific emotional states: Cordaro et al., 2018), and thereafter instruct participants to express their emotions in front of a video camera. While excellent experimental control can be achieved with this setup, a key drawback is low ecological validity (e.g., white room effects: Cicourel, 1996). In contrast, naturalistic approaches focus on capturing emotional expressions as they occur in people’s everyday life (e.g., at the exact moment when an athlete is victorious: Tracy & Matsumoto, 2008). While high in ecological validity, a lack of experimental control can limit the conclusions that can be drawn from the data. Specifically, researchers are required to assume that specific emotional states are being experienced in these situations (as self-report measurement is often impractical), and because most data collection is opportunistic, the role of incidental environmental factors in shaping emotional expressions cannot be disentangled from the influence of the elicited emotion per se. The same issue applies to self-selection biases regarding ecologically valid situations; the victorious in a contest may differ from those who were defeated on a variety of other characteristics (e.g., personality: Piepiora, 2021), and these influences are difficult to rule out without random assignment. Here, we opted for an alternative approach that contends with the tension between experimental control and ecological validity: the participant-negotiated recall method (Madore et al., 2019).

**Supplement B: Emotion Definitions**

**Amusement**

The feeling when you encounter something silly, ironic, witty, or absurd, which makes you laugh. You have the urge to be playful and share the joke with others.

**Awe**

The feeling when you encounter something that is greater or more powerful than yourself. You feel overwhelmed and need a moment to adjust.

**Interest**

The feeling when you encounter something new and relevant that you do not immediately understand. You have the urge to find out more about it.

**Relief**

The feeling when an unpleasant experience is finally over, or when you find out that something you had dreaded has not happened (or will not happen). You can finally take your mind off it.

**Positive Surprise**

The feeling when you realize something good or nice just happened, which you did not expect. You need a moment to take in the good news.

**Tenderness**

The feeling when you encounter someone or something that seems cute, vulnerable, or childlike in appearance or behaviour. You have the urge to nurture and care for this person or thing.

**Gratitude**

The feeling when you think that someone has gone out of their way to do something good or nice for you. You have the urge to do something back and get closer to this person.

**Admiration**

The feeling when you look up to someone who has excellent abilities or has accomplished impressive things. You have the urge to also achieve such things and be more like this person.

**Sexual Desire**

The feeling when you think about or interact with someone that you find sexually attractive. You have the urge to be physically close and have sexual relations with this person.

**Sensory Pleasure**

The feeling when something happens that pleases your senses.

**Schadenfreude**

The feeling when something bad happens to another person. You enjoy this because you dislike the person, because you think the person deserves it, or because it is somehow good for you.

**Elation**

The feeling when something very good just happened to you. You have the urge to celebrate and share it with others.

**Pride**

The feeling when you (or someone close to you) possess or have accomplished something that other people find praiseworthy.

**Triumph**

The feeling of release and great joy after the successful ending of a struggle or contest.

**Inspiration**

The feeling when you suddenly get a new idea or insight, or see the world in a different light. You have the urge to express or actualise this new insight.

**Determination**

The feeling when you are working on a demanding problem or task that you believe you can handle. You feel stimulated to accomplish it.

**Hope**

The feeling when you believe that a desired event has a possibility of happening. You keep thinking about how good it would be if the event actually happened.

**Elevation**

The feeling when you witness someone’s noble or good deed. You have the urge to praise this person and to also do good yourself.

**Amae**

The feeling when you think about or interact with a person or entity to whose love, care and protection you want to submit yourself.

**Moved**

The feeling when you encounter something very beautiful, meaningful, or bittersweet. Tears well up in your eyes and you feel overcome with warm feelings.

**Excitement**

The feeling when you expect something good or nice will happen to you. You cannot stop thinking about this.

**Respected**

The feeling when you are valued, recognised and deemed worthy by others, even when they may disagree with your opinions.

**Supplement C: Additional Details about the FACS**

The Facial Action Coding System (FACS) is recognized as an established method in the field of psychological science (Ekman, 1982). In this system, each facial behavior (e.g., lip corner puller) is assigned a code (e.g., AU12). The validity and reliability of the FACS has been rigorously tested by robust lines of inquiry, and it has been adapted for use in a variety of applied contexts that go beyond psychological research (Rosenberg & Ekman, 2020). The FACS was first developed to be manually applied by human coders (with certification requirements that involve passing an examination), and hence requires substantial time investment when used for research purposes. However, in recent years, automated methods that are based on deep learning algorithms building on FACS have been made available. The degree to which such software coheres with human coders remains inconclusive, though the accuracy of these algorithms has increased with updated versions (for a comparison of tools, see Dupre et al., 2020). Machine based FACS coding is mostly applied in spontaneous interactions, mainly for pragmatic reasons involving ease of use when a dataset is large. In such cases, the norm is to apply manual FACS coding to a portion of the data and evaluate convergent validity before any further analysis is conducted. Given our focus on spontaneous expressions and the size of our dataset, we adopted this approach. Table S1 shows an overview of comparisons between the manual and machine based FACS coding based on relevant indices.

**Table S1**

*Estimates of Convergent Validity*

| Action Unit | Description | Presence in Counts | | Recall | Precision | F1 | |
| --- | --- | --- | --- | --- | --- | --- | --- |
|  |  | FaceReader | FACS |  |  | Index | Threshold |
| AU1 | Inner Brow Raise | 148 | 138 | 0.76 | 0.71 | 0.73 | **✓** |
| AU2 | Outer Brow Raise | 119 | 143 | 0.62 | 0.75 | 0.68 | **✓** |
| AU4 | Brow Lowerer | 218 | 32 | 0.91 | 0.13 | 0.23 | **✕** |
| AU5 | Upper Lid Raise | 56 | 103 | 0.2 | 0.38 | 0.26 | **✕** |
| AU6 | Cheek Raise | 185 | 110 | 0.92 | 0.55 | 0.69 | **✓** |
| AU7 | Lid Tightener | 186 | 212 | 0.68 | 0.77 | 0.72 | **✓** |
| AU9 | Nose Wrinkle | 60 | 1 | 1 | 0.02 | 0.04 | **✕** |
| AU10 | Upper Lip Raiser | 183 | 12 | 0.75 | 0.05 | 0.09 | **✕** |
| AU12 | Lip Corner Puller | 199 | 148 | 0.91 | 0.68 | 0.78 | **✓** |
| AU14 | Dimpler | 47 | 0 | NA | 0 | NA | **✕** |
| AU15 | Lip Corner Depressor | 44 | 9 | 0.11 | 0.02 | 0.03 | **✕** |
| AU17 | Chin Raiser | 201 | 241 | 0.76 | 0.91 | 0.83 | **✓** |
| AU18 | Lip Pucker | 54 | 32 | 0.28 | 0.17 | 0.21 | **✕** |
| AU20 | Lip Stretch | 77 | 32 | 0.38 | 0.16 | 0.23 | **✕** |
| AU23 | Lip Tightener | 41 | 22 | 0.14 | 0.07 | 0.09 | **✕** |
| Action Unit | Description | Presence in Counts | | Recall | Precision | F1 | |
|  |  | FaceReader | FACS |  |  | Index | Threshold |
| AU24 | Lip Presser | 115 | 129 | 0.43 | 0.48 | 0.45 | **✕** |
| AU25 | Lips Part | 254 | 264 | 0.96 | 1 | 0.98 | **✓** |
| AU26 | Jaw Drop | 237 | 264 | 0.9 | 1 | 0.95 | **✓** |
| AU27 | Mouth Stretch | 57 | 56 | 0.29 | 0.28 | 0.28 | **✕** |
| AU43 | Eye Closure | 203 | 208 | 0.78 | 0.8 | 0.79 | **✓** |
| AU45 | Blink | 106 | 135 | 0.34 | 0.43 | 0.38 | **✕** |
| AU51 | Head Turn Left | 233 | 104 | 0.98 | 0.44 | 0.61 | **✕** |
| AU52 | Head Turn Right | 237 | 125 | 0.96 | 0.51 | 0.67 | **✓** |
| AU53 | Head Up | 262 | 71 | 0.99 | 0.27 | 0.42 | **✕** |
| AU54 | Head Down | 88 | 100 | 0.46 | 0.52 | 0.49 | **✕** |

*Note.* Ticks (crosses) denote acceptable (unacceptable) indices. AUs that did not meet the preset threshold for validity were excluded.

**Supplement D: Self-reported emotional experience**

163 participants were recruited, of which 137 reported Dutch as their first language, 26 reported to be bilingual, for whom first language included, in addition to Dutch: English (*N* = 11), German (*N* = 2), Frisian (*N* = 2), Bengal (*N* = 1), Berber (*N* = 1), French (*N* = 1), Greek (*N* = 1), Italian (*N* = 1), Polish (*N* = 1), Russian (*N* = 1), Spanish (*N* = 1), Turkish (*N* = 1), and Swedish (*N* = 1). 117 participants reported their ethnicity as “Dutch”, 19 as “White/Caucasian”, 3 as “Moroccan”, 1 as “Belgian”, 1 as “Chinese”, 1 as “Hindu”, 1 as “Northern European”, 1 as “Black”, 1 as “Indian”, 1 as “Caribbean Dutch”. 20 participants reported being of mixed descendance: American/Dutch (*N* = 1), Asian/White (*N* = 2), British/Dutch (*N* = 1), Indonesian/Dutch (*N* = 2), Congolese/Dutch (*N* = 1), Tunesian/Dutch (*N* = 1), German/Dutch (*N* = 1), Ecuadorian/Dutch (*N* = 1), Turkish/Dutch (*N* = 2), Jamaican/Dutch (*N* = 1), Surinam/Dutch (*N* =2), Nepalese/Hungarian (*N* = 1), Nigerian/Dutch (*N* = 1).

**Supplement E: Self-reported emotional experience**

In additional to facial expressions, we collected self-report measures of perceived social engagement, emotion intensity during the episode and recall, valence, and physiological arousal to gain a broader understanding of people’s emotion experiences. For each emotion, participants answered questions relating to their experience of the target emotion (administered via Qualtrics). We first measured perceived social engagement, by asking participants how connected they felt with others in their life during the situations in which they experienced each emotion (7 stages of overlapping circles: see Aron et al., 1992). Three manipulation check items followed. We measured how intense the experience of the emotion was (1 = *not at all*, 7 = *very much*), first during recall, and then at the time of the actual event, and next measured experience valence during recall (1 = *not at all positive*, 7 = *very positive*). Finally, we included an item measuring physiological arousal experienced during recall (*1 =* *low arousal – very passive, drowsy, slow heart rate, slow breathing*, 7 = *high arousal – very active, increased heart rate, accelerated breathing, sweaty palms*).

We first examined if our paradigm successfully elicited the intended positive emotions. For all 22 positive emotions, self-reported intensity of the target emotion during the episode was recorded to be significantly above the scale midpoint (range from *1* to *7*; also see Figure S1a: *t*s > 11.41, *p*s < .001, Cohen’s *d*s > .92). This indicates that participants’ selected episodes induced the emotions as intended (all *M*s > 5.27). For intensity during recall, emotions were reported to be experienced at levels that were significantly above midpoint as well (Figure S1b: *t*s > 4.25, *p*s < .001, Cohen’s *d*s > .33), with the exceptions of sexual desire (*M* = 4.01, *SD* = 1.48), schadenfreude (*M* = 4.31, *SD* = 1.53), and sensory pleasure (*M* = 4.35, *SD* = 1.56). Even during recall, most of the emotion episodes thus elicited the intended emotions fairly intensely. The experience of schadenfreude, sexual desire, and sensory pleasure may be strongly tied to situational elicitors, which may explain why intensity was weaker during recall.

As for experienced valence, apart from schadenfreude (*M* = 4.08, *SD* = 1.26), all emotions were rated to be significantly more positive than the scale midpoint (Figure S1c: *t*s > 10.92, *p*s < .001, Cohen’s *d*s > .86), suggesting that participants felt pleasant when recalling each of the emotions. Schadenfreude is suggested to be an ambivalent emotion (Smith et al., 2009), meaning that participants positive feelings of malicious glee could have been counteracted against by negative feelings of guilt at taking joy in another’s downfall, thereby leading to an average score that was slightly below scale midpoint.

When attempting to understand how these emotions relate to one another, researchers have tried to taxonomize positive emotions based on specific features, such as physiological arousal (Tsai et al., 2006) and social engagement (Kitayama et al., 2006). For example, cultural psychologists have proposed arousal and social engagement as underlying dimensions: how physiologically arousing a positive emotion is experienced as feeling (e.g., heightened or diminished bodily responses: Tsai et al., 2006) or how socially engaging its experience is deemed to be (e.g., facilitates closeness with others versus withdrawal towards the self: Kitayama et al., 2006). These dimensions are orthogonal and complementary, such that they can be applied in combination with each other to classify positive emotions (also see Sun et al., 2021). We hence measured both self-reported social engagement and physiological arousal per positive emotion.

The emotions differed substantially with regards to social engagement, *F*(21, 3357.8) = 82.05, *p* < .001, *r*^2^ = .336, and to a lesser degree self-reported physiological arousal, *F*(21, 3357.2) = 16.99, *p* < .001, *r*^2^ = .096. Findings align with previous data (e.g., Sun et al., 2021) on engagement and arousal levels of various positive emotions (see Figure S2). For example, as expected, determination and sensory pleasure were rated to be more self-focused, while gratitude and amae were found to be more socially oriented. Differences in physiological arousal were smaller, but also consistent with earlier work: excitement and sexual desire was rated to be relatively high, while moral elevation and feeling respected were found to be relatively low. Taken together, these findings provide confidence in the efficacy of the participant negotiated recall method to elicit the target emotions. Yet we also acknowledge that in addition to the target emotion, participants could have been experiencing other emotions when narrating stories (e.g., mixed emotions: Larsen & McGraw, 2014). However, it was simply unfeasible to include multi-item experience measures in each condition.

**Figure S1**

*Results For Event Intensity, Recall Intensity and Self-Reported Valence*

*Note.* Event and recall intensity range from 1 = low intensity; 7 = high intensity and self-reported valence ranges from 1 = negative; 7 = positive. Dashed line indicates scale midpoint. Dots represent means and error bars represent standard errors.

**Figure S2**

*Pattern of Results for Self-Reported Physiological Arousal and Social Engagement*

*Note*. The Y-axis has been truncated for clarity, as differences across emotions in reported arousal were too small to chart at original scale.

**Supplement F: Inferential and Descriptive Statistics for Action Units**

Based on past research and theorizing, we expected particular action units to be activated for each specific positive emotion (e.g., AUs 6, 7, 12 and 43 for being moved: see Table 1 below). Hypotheses were pre-registered for the predicted expressions of each positive emotion: <https://osf.io/qb3mc/?view_only=f0832ed57d5447c2ad6803279375f784>

**Table S1**

*Literature and Hypotheses Overview*

| Emotion | Relevant Citations | Predicted Facial Behaviours (and related Action Units) |
| --- | --- | --- |
| Admiration | *Onu et al., 2016*; Bänziger et al., 2012 | Lip Corner Puller (AU12), Lip Presser (AU24), Head Up (AU53) |
| Amae | *Niiya et al., 2000* | Cheek Raiser (AU6), Lip Corner Puller (AU12), Head Down (AU54) |
| Amusement | Campos et al., 2013; Shiota et al., 2003;  Cordaro et al., 2018; **Ambadar et al., 2009** | Cheek Raiser (AU6), Lid Tightener (AU7), Lip Corner Puller (AU12), Lower Lip Depressor (AU16), Lips Part (AU25), Jaw Drop (AU26), Head Up (AU53) |
| Awe | *Monroy & Keltner, 2022*; Shiota et al., 2003;  Campos et al., 2013; Cordaro et al., 2018 | Inner Brow Raiser (AU1), Outer Brow Raiser (AU2), Upper Lid Raiser (AU5), Lips Part (AU25), Jaw Drop (AU26), Head Up (AU53) |
| Being Moved | *Zickfeld et al., 2019*; Schubert et al., 2018 | Cheek Raiser (AU6), Lid Tightener (AU7), Lip Corner Puller (AU12), Eye Closure (AU43) |
| Determination | **Rozin & Cohen, 2003** | Inner Brow Raiser (AU1), Brow Lowerer (AU4), Lid Tightener (AU7), Lip Presser (AU24) |
| Elation | Mortillaro et al., 2011; Ricci-Bitti et al., 1996; Cordaro et al., 2018; **Wallbott, 1998, Dael et al., 2012** | Inner Brow Raiser (AU1), Outer Brow Raiser (AU2), Cheek Raiser (AU6), Lip Corner Puller (AU12), Chin Raiser (AU17), Lips Part (AU25), Jaw Drop (AU26) |
| Excitement | *Stratton, 1928; Lyng, 2018* | Inner Brow Raiser (AU1), Outer Brow Raiser (AU2), Cheek Raiser (AU6), Lip Corner Puller (AU12), Lips Part (AU25), Jaw Drop (AU26) |
| Feeling Respected | *Li & Fischer, 2007* | Cheek Raiser (AU6), Lid Tightener (AU7), Lip Corner Puller (AU12), Lip Pressor (AU24), Head Up (AU53) |
| Gratitude | *Algoe & Zhaoyang, 2016*; Campos et al., 2013 | Lip Corner Puller (AU12), Head Down (AU54) |
| Hope | *Lazarus, 1999*; Cohen-chen et al., 2019 | Inner Brow Raiser (AU1), Outer Brow Raiser (AU2), Cheek Raiser (AU6), Lip Corner Puller (AU12), Chin Raiser (AU17), Head Up (AU53) |
| Interest | *Silvia, 2008*; Mortillaro et al., 2011; Reeve, 1993; Campos et al., 2013; Cordaro et al., 2018 | Inner Brow Raiser (AU1), Brow Lowerer (AU4), Head Up (AU53), Head Down (AU54) |
| Inspiration | *Thrash & Elliot, 2003; Thrash et al., 2014* | Inner Brow Raiser (AU1), Lips Part (AU25), Lip Corner Puller (AU12) |
| Moral Elevation | *Thomson & Siegel, 2017; Schnall & Roper, 2012* | Cheek Raiser (AU6), Lip Corner Puller (AU12), Lips Parting (AU25),  Jaw Drop (AU26), Head Up (AU53) |
| Positive Surprise | Vrticka et al., 2014; Cordaro et al., 2018 | Inner Brow Raiser (AU1), Outer Brow Raiser (AU2), Upper Lid Raiser (AU5), Cheek Raiser (AU6), Lip Corner Puller (AU12), Jaw Drop (AU26), Lips Part (AU25) |
| Pride | Mortillaro et al., 2011; Cordaro et al., 2018;  **Tracy & Matsumoto, 2008**; **Tracy & Robins, 2004** | Lid Tightener (AU7), Lip Corner Puller (AU12), Lip Pressor (AU24), Head Up (AU53) |
| Relief | Cordaro et al., 2018; **Krumhuber & Scherer, 2011** | Lip Corner Puller (AU12), Lips Pucker (AU18), Lips Part (AU25), Jaw Drop (AU26), Eye Closure (AU43), Head Up (AU53), Head Down (AU54) |
| Schadenfreude | Rychlowska et al., 2017; **Boecker et al., 2015** | Brow Lowerer (AU4), Cheek Raiser (AU6), Lip Corner Puller (AU12) |
| Sensory Pleasure | Ricci-Bitti et al., 1996; Mortillaro et al., 2011;  Cordaro et al., 2018; **Fernandez-Dols et al., 2011** | Cheek Raiser (AU6), Lid Tightener (AU7), Upper Lip Raiser (AU10),  Lip Corner Puller (AU12), Lips Part (AU25), Jaw Drop (AU26), Eye Closure (AU43) |
| Sexual Desire | Cordaro et al., 2018; **Gonzaga et al., 2006;**  **Fernandez-Dols et al., 2011** | Cheek Raiser (AU6), Lid Tightener (AU7), Lip Corner Puller (AU12),  Lip Pucker (AU18), Lips Part (AU25) |
| Tenderness | Campos et al., 2013; **Gonzaga et al., 2001** | Cheek Raiser (AU6), Lip Corner Puller (AU12) |
| Triumph | Cordaro et al., 2018; **Hwang & Matsumoto, 2014; Kalokerinos et al., 2014** | Cheek Raiser (AU6), Lid Tightener (AU7), Lip Corner Puller (AU12),  Lips Part (AU25), Jaw Drop (AU26), Head Up (AU53) |

*Note*. List of emotions, studies on which the hypotheses were based, and predictions about specific facial behaviors. Bold denotes evidence from spontaneous expressions, regular text indicates studies of posed expressions, and italics refer to theoretical proposals.

We then tested these predictions with classical frequency analyses (i.e., nonparametric chi-square tests) to examine whether the occurrence rate of a hypothesized AU was higher than chance level for that given emotion. Frequency analyses are the most commonly used method in the study of emotion expression production, used by researchers to chart out how often different facial behaviours occur during different emotions. In this approach, inferences are drawn from raw occurrence rates, based on how frequently an AU occurs for a given emotion. For example, an occurrence rate between 25% to 49% can be thought of as a weak association between a given AU and an emotion, while frequencies that are larger than 50% can be classified as a moderate association (Campos et al., 2013). Hypothesis testing is conducted with non-parametric frequency tests using highly conservative chance thresholds (e.g., Cordaro et al., 2018) that attempt to mitigate against false positives (see Elfenbein et al., 2007).

For each emotion separately, we tested whether the occurrence of the hypothesized AU(s) was significantly above chance level using non-parametric *t*-tests. With 10 AUs, the probability of a given facial behavior occurring purely by chance was 10%. Yet following the recommendation of prior work, which suggests that six upper-face AUs mostly drive facial expressions during emotional episodes (see Elfenbein et al., 2007; Cordaro et al., 2018), we applied a more conservative chance level of 16.7% (i.e., 1/6). Thereby, we tested whether the occurrence of an AU was significantly higher than 16.7% of the time, while applying a Bonferroni-correction resulting in *α* = .0002.

Using this conservative threshold, the results demonstrated support for all of our hypotheses regarding these 10 Action Units. Table S2 provides an overview of the results of these non-parametric tests per emotion and Table S3-S6 provide an overview of the raw frequencies of each AU across all emotion expressions. We further summarize our findings for both the non-parametric chi-square tests and the network results in Table S7, which shows whether each hypothesized AU was found to occur significantly. It also provides an overview of raw occurrence rates of each AU per emotion by classifying them into rank ordered categories (< 25%, from 25% to 50%, from 50% to 75%, and > 75%) following previous work (Campos et al., 2013). For example, feeling respected was found to be expressed with AUs 6, 7, and 12, and all of these behaviors occurred for more than 50% of participants (as denoted by asterisks in Table S7), meaning that a majority of our sample produced these facial movements when recollecting an experience of feeling respected. In fact, for all emotions, all these 10 AUs occurred significantly more often than would be expected by chance (*χ^2^*s > 20.85, *p*s < .001, Cohen’s *h*s > .33).

Although the fixed thresholds used here were corrected and hence meant to be conservative (Elfenbein et al., 2007), these analyses do not account for the fact that some AUs generally occur more (or less) frequently, regardless of emotion. For example, frequent activation of AUs 25 (Lip Part) and 26 (Jaw Drop) across positive emotions may reflect the fact that participants were talking. While fixed threshold comparisons are not designed to provide an index of specificity, these findings provide an overview of which AUs occur per emotion.

Together, Table S7 provides an overview of results from the non-parametric chi-square tests and network models respectively, allowing for a direct comparison between the findings from both analytic methods. Two clear patterns of results emerge. First, in our non-parametric chi-square analyses, all 10 Action Units were found to occur at significantly higher rates than chance (even after applying a conservative threshold) across the set of 22 positive emotions. Each included AU occurred at least 30.4% in frequency per positive emotion, hence explaining this finding. We interpret this result to mean that spontaneous positive emotional expressions in general may include a multitude of facial behaviors that occur quite frequently.

**Table S2**

*Results for Non-Parametric t-tests*

| Emotion | AU | *χ*2 | 95% CI | Cohen’s *h* |
| --- | --- | --- | --- | --- |
| Admiration | AU01 | 141.38 | [0.45, 1] | 0.77 |
|  | AU02 | 52.65 | [0.32, 1] | 0.49 |
|  | AU06 | 287.17 | [0.6, 1] | 1.07 |
|  | AU07 | 316.43 | [0.63, 1] | 1.12 |
|  | AU12 | 331.60 | [0.64, 1] | 1.15 |
|  | AU17 | 355.01 | [0.66, 1] | 1.19 |
|  | AU25 | 743.53 | [0.93, 1] | 1.95 |
|  | AU26 | 622.54 | [0.85, 1] | 1.66 |
|  | AU43 | 412.75 | [0.7, 1] | 1.29 |
|  | AU53 | 778.40 | [0.96, 1] | 2.08 |
| Amae | AU43 | 357.69 | [0.66, 1] | 1.21 |
|  | AU53 | 763.44 | [0.96, 1] | 2.07 |
| Amusement | AU01 | 122.05 | [0.43, 1] | 0.72 |
|  | AU02 | 55.75 | [0.33, 1] | 0.50 |
|  | AU06 | 612.07 | [0.85, 1] | 1.64 |
|  | AU07 | 412.75 | [0.7, 1] | 1.29 |
|  | AU12 | 601.69 | [0.84, 1] | 1.62 |
|  | AU17 | 323.97 | [0.63, 1] | 1.13 |
|  | AU25 | 766.69 | [0.95, 1] | 2.03 |
|  | AU26 | 622.54 | [0.85, 1] | 1.66 |
|  | AU43 | 430.05 | [0.72, 1] | 1.32 |
|  | AU53 | 766.69 | [0.95, 1] | 2.03 |
| Awe | AU01 | 155.23 | [0.47, 1] | 0.80 |
|  | AU02 | 51.27 | [0.32, 1] | 0.49 |
|  | AU06 | 300.48 | [0.61, 1] | 1.10 |
|  | AU07 | 370.25 | [0.67, 1] | 1.22 |
|  | AU12 | 386.75 | [0.69, 1] | 1.25 |
|  | AU17 | 330.59 | [0.64, 1] | 1.15 |
|  | AU25 | 745.10 | [0.94, 1] | 1.98 |
|  | AU26 | 602.26 | [0.84, 1] | 1.64 |
|  | AU43 | 395.13 | [0.69, 1] | 1.27 |
|  | AU53 | 780.22 | [0.97, 1] | 2.14 |
| Being Moved | AU01 | 88.87 | [0.38, 1] | 0.62 |
|  | AU02 | 20.86 | [0.25, 1] | 0.33 |
|  | AU06 | 312.15 | [0.62, 1] | 1.12 |
|  | AU07 | 319.66 | [0.63, 1] | 1.13 |
|  | AU12 | 366.60 | [0.67, 1] | 1.21 |
|  | AU17 | 297.39 | [0.61, 1] | 1.09 |
| Emotion | AU | *χ*2 | 95% CI | Cohen’s *h* |
| Being Moved | AU25 | 738.55 | [0.93, 1] | 1.95 |
|  | AU26 | 566.23 | [0.82, 1] | 1.56 |
|  | AU43 | 434.19 | [0.72, 1] | 1.33 |
|  | AU53 | 761.70 | [0.95, 1] | 2.03 |
| Determination | AU01 | 117.44 | [0.42, 1] | 0.70 |
|  | AU02 | 58.94 | [0.33, 1] | 0.52 |
|  | AU06 | 226.51 | [0.54, 1] | 0.95 |
|  | AU07 | 339.32 | [0.64, 1] | 1.16 |
|  | AU12 | 266.15 | [0.58, 1] | 1.03 |
|  | AU17 | 379.23 | [0.68, 1] | 1.23 |
|  | AU25 | 755.07 | [0.94, 1] | 1.98 |
|  | AU26 | 612.07 | [0.85, 1] | 1.64 |
|  | AU43 | 438.83 | [0.72, 1] | 1.33 |
|  | AU53 | 790.20 | [0.97, 1] | 2.14 |
| Elation | AU01 | 117.44 | [0.42, 1] | 0.70 |
|  | AU02 | 35.91 | [0.28, 1] | 0.42 |
|  | AU06 | 447.70 | [0.73, 1] | 1.35 |
|  | AU07 | 387.48 | [0.68, 1] | 1.25 |
|  | AU12 | 502.78 | [0.77, 1] | 1.44 |
|  | AU17 | 339.32 | [0.64, 1] | 1.16 |
|  | AU25 | 778.40 | [0.96, 1] | 2.08 |
|  | AU26 | 643.74 | [0.87, 1] | 1.70 |
|  | AU43 | 421.36 | [0.71, 1] | 1.30 |
|  | AU53 | 790.20 | [0.97, 1] | 2.14 |
| Elevation | AU01 | 194.40 | [0.51, 1] | 0.89 |
|  | AU02 | 45.38 | [0.31, 1] | 0.46 |
|  | AU06 | 200.36 | [0.52, 1] | 0.90 |
|  | AU07 | 315.35 | [0.63, 1] | 1.13 |
|  | AU12 | 218.77 | [0.54, 1] | 0.94 |
|  | AU17 | 429.55 | [0.72, 1] | 1.33 |
|  | AU25 | 733.57 | [0.93, 1] | 1.94 |
|  | AU26 | 512.27 | [0.78, 1] | 1.47 |
|  | AU43 | 354.12 | [0.66, 1] | 1.20 |
|  | AU53 | 780.22 | [0.97, 1] | 2.14 |
| Excitement | AU01 | 122.05 | [0.43, 1] | 0.72 |
|  | AU02 | 65.59 | [0.34, 1] | 0.54 |
|  | AU06 | 447.70 | [0.73, 1] | 1.35 |
|  | AU07 | 371.07 | [0.67, 1] | 1.22 |
|  | AU12 | 474.84 | [0.75, 1] | 1.39 |
|  | AU17 | 308.98 | [0.62, 1] | 1.11 |
|  | AU25 | 766.69 | [0.95, 1] | 2.03 |
|  | AU26 | 633.09 | [0.86, 1] | 1.68 |
|  | AU43 | 355.01 | [0.66, 1] | 1.19 |
|  | AU53 | 790.20 | [0.97, 1] | 2.14 |
| Gratitude | AU01 | 182.22 | [0.5, 1] | 0.86 |
|  | AU02 | 90.42 | [0.38, 1] | 0.62 |
| Emotion | AU | *χ*2 | 95% CI | Cohen’s *h* |
| Gratitude | AU06 | 284.22 | [0.6, 1] | 1.06 |
|  | AU07 | 335.95 | [0.64, 1] | 1.15 |
|  | AU12 | 298.56 | [0.61, 1] | 1.09 |
|  | AU17 | 383.72 | [0.68, 1] | 1.23 |
|  | AU25 | 748.51 | [0.93, 1] | 1.95 |
|  | AU26 | 596.28 | [0.83, 1] | 1.60 |
|  | AU43 | 434.68 | [0.72, 1] | 1.32 |
|  | AU53 | 795.18 | [0.97, 1] | 2.14 |
| Hope | AU01 | 167.54 | [0.48, 1] | 0.83 |
|  | AU02 | 72.59 | [0.35, 1] | 0.57 |
|  | AU06 | 301.62 | [0.61, 1] | 1.09 |
|  | AU07 | 339.32 | [0.64, 1] | 1.16 |
|  | AU12 | 387.48 | [0.68, 1] | 1.25 |
|  | AU17 | 294.35 | [0.61, 1] | 1.08 |
|  | AU25 | 720.74 | [0.92, 1] | 1.88 |
|  | AU26 | 633.09 | [0.86, 1] | 1.68 |
|  | AU43 | 430.05 | [0.72, 1] | 1.32 |
|  | AU53 | 778.40 | [0.96, 1] | 2.08 |
| Inspiration | AU01 | 226.51 | [0.54, 1] | 0.95 |
|  | AU02 | 79.95 | [0.37, 1] | 0.59 |
|  | AU06 | 214.00 | [0.53, 1] | 0.93 |
|  | AU07 | 316.43 | [0.63, 1] | 1.12 |
|  | AU12 | 331.60 | [0.64, 1] | 1.15 |
|  | AU17 | 347.12 | [0.65, 1] | 1.18 |
|  | AU25 | 698.29 | [0.9, 1] | 1.82 |
|  | AU26 | 591.40 | [0.83, 1] | 1.60 |
|  | AU43 | 387.48 | [0.68, 1] | 1.25 |
|  | AU53 | 766.69 | [0.95, 1] | 2.03 |
| Interest | AU01 | 146.43 | [0.46, 1] | 0.78 |
|  | AU02 | 43.88 | [0.3, 1] | 0.45 |
|  | AU06 | 245.93 | [0.56, 1] | 0.99 |
|  | AU07 | 287.17 | [0.6, 1] | 1.07 |
|  | AU12 | 323.97 | [0.63, 1] | 1.13 |
|  | AU17 | 331.60 | [0.64, 1] | 1.15 |
|  | AU25 | 732.09 | [0.93, 1] | 1.91 |
|  | AU26 | 591.40 | [0.83, 1] | 1.60 |
|  | AU43 | 438.83 | [0.72, 1] | 1.33 |
|  | AU53 | 778.40 | [0.96, 1] | 2.08 |
| Positive Suprise | AU01 | 158.67 | [0.47, 1] | 0.81 |
|  | AU02 | 101.22 | [0.4, 1] | 0.66 |
|  | AU06 | 425.43 | [0.71, 1] | 1.31 |
|  | AU07 | 297.39 | [0.61, 1] | 1.09 |
|  | AU12 | 451.98 | [0.73, 1] | 1.36 |
|  | AU17 | 312.15 | [0.62, 1] | 1.12 |
|  | AU25 | 750.08 | [0.94, 1] | 1.98 |
|  | AU26 | 596.80 | [0.84, 1] | 1.62 |
| Emotion | AU | *χ*2 | 95% CI | Cohen’s *h* |
| Positive Surprise | AU43 | 461.01 | [0.74, 1] | 1.38 |
|  | AU53 | 785.21 | [0.97, 1] | 2.14 |
| Pride | AU01 | 118.94 | [0.42, 1] | 0.71 |
|  | AU02 | 66.57 | [0.35, 1] | 0.55 |
|  | AU06 | 290.15 | [0.6, 1] | 1.08 |
|  | AU07 | 334.95 | [0.64, 1] | 1.16 |
|  | AU12 | 382.96 | [0.68, 1] | 1.24 |
|  | AU17 | 391.28 | [0.69, 1] | 1.26 |
|  | AU25 | 727.11 | [0.93, 1] | 1.91 |
|  | AU26 | 596.80 | [0.84, 1] | 1.62 |
|  | AU43 | 319.66 | [0.63, 1] | 1.13 |
|  | AU53 | 785.21 | [0.97, 1] | 2.14 |
| Relief | AU01 | 165.97 | [0.48, 1] | 0.83 |
|  | AU02 | 71.10 | [0.35, 1] | 0.57 |
|  | AU06 | 346.19 | [0.65, 1] | 1.18 |
|  | AU07 | 370.25 | [0.67, 1] | 1.22 |
|  | AU12 | 346.19 | [0.65, 1] | 1.18 |
|  | AU17 | 386.75 | [0.69, 1] | 1.25 |
|  | AU25 | 710.79 | [0.92, 1] | 1.88 |
|  | AU26 | 581.63 | [0.83, 1] | 1.60 |
|  | AU43 | 429.55 | [0.72, 1] | 1.33 |
|  | AU53 | 780.22 | [0.97, 1] | 2.14 |
| Respected | AU01 | 136.42 | [0.45, 1] | 0.75 |
|  | AU02 | 49.64 | [0.31, 1] | 0.48 |
|  | AU06 | 156.81 | [0.47, 1] | 0.80 |
|  | AU07 | 331.60 | [0.64, 1] | 1.15 |
|  | AU12 | 162.13 | [0.48, 1] | 0.82 |
|  | AU17 | 371.07 | [0.67, 1] | 1.22 |
|  | AU25 | 755.07 | [0.94, 1] | 1.98 |
|  | AU26 | 591.40 | [0.83, 1] | 1.60 |
|  | AU43 | 395.81 | [0.69, 1] | 1.26 |
|  | AU53 | 778.40 | [0.96, 1] | 2.08 |
| Schadenfreude | AU01 | 77.63 | [0.37, 1] | 0.60 |
|  | AU02 | 24.23 | [0.26, 1] | 0.36 |
|  | AU06 | 419.34 | [0.72, 1] | 1.34 |
|  | AU07 | 304.61 | [0.63, 1] | 1.13 |
|  | AU12 | 464.75 | [0.76, 1] | 1.42 |
|  | AU17 | 297.12 | [0.62, 1] | 1.11 |
|  | AU25 | 692.28 | [0.92, 1] | 1.90 |
|  | AU26 | 614.99 | [0.87, 1] | 1.71 |
|  | AU43 | 401.83 | [0.71, 1] | 1.31 |
|  | AU53 | 738.51 | [0.96, 1] | 2.07 |
| Sensory Pleasure | AU01 | 146.43 | [0.46, 1] | 0.78 |
|  | AU02 | 65.59 | [0.34, 1] | 0.54 |
|  | AU06 | 456.66 | [0.74, 1] | 1.36 |
|  | AU07 | 465.71 | [0.74, 1] | 1.38 |
| Emotion | AU | *χ*2 | 95% CI | Cohen’s *h* |
| Sensory Pleasure | AU12 | 512.28 | [0.78, 1] | 1.46 |
|  | AU17 | 347.12 | [0.65, 1] | 1.18 |
|  | AU25 | 766.69 | [0.95, 1] | 2.03 |
|  | AU26 | 622.54 | [0.85, 1] | 1.66 |
|  | AU43 | 430.05 | [0.72, 1] | 1.32 |
|  | AU53 | 790.20 | [0.97, 1] | 2.14 |
| Sexual Desire | AU01 | 123.56 | [0.43, 1] | 0.73 |
|  | AU02 | 38.56 | [0.29, 1] | 0.43 |
|  | AU06 | 353.24 | [0.66, 1] | 1.20 |
|  | AU07 | 329.60 | [0.64, 1] | 1.16 |
|  | AU12 | 329.60 | [0.64, 1] | 1.16 |
|  | AU17 | 284.77 | [0.6, 1] | 1.08 |
|  | AU25 | 723.62 | [0.93, 1] | 1.94 |
|  | AU26 | 602.89 | [0.85, 1] | 1.65 |
|  | AU43 | 446.94 | [0.74, 1] | 1.37 |
|  | AU53 | 758.45 | [0.96, 1] | 2.07 |
| Tenderness | AU01 | 99.89 | [0.4, 1] | 0.65 |
|  | AU02 | 28.73 | [0.27, 1] | 0.38 |
|  | AU06 | 474.84 | [0.75, 1] | 1.39 |
|  | AU07 | 347.12 | [0.65, 1] | 1.18 |
|  | AU12 | 465.71 | [0.74, 1] | 1.38 |
|  | AU17 | 273.06 | [0.59, 1] | 1.04 |
|  | AU25 | 732.09 | [0.93, 1] | 1.91 |
|  | AU26 | 601.69 | [0.84, 1] | 1.62 |
|  | AU43 | 438.83 | [0.72, 1] | 1.33 |
|  | AU53 | 778.40 | [0.96, 1] | 2.08 |
| Triumph | AU01 | 141.38 | [0.45, 1] | 0.77 |
|  | AU02 | 65.59 | [0.34, 1] | 0.54 |
|  | AU06 | 421.36 | [0.71, 1] | 1.30 |
|  | AU07 | 294.35 | [0.61, 1] | 1.08 |
|  | AU12 | 484.07 | [0.76, 1] | 1.41 |
|  | AU17 | 301.62 | [0.61, 1] | 1.09 |
|  | AU25 | 743.53 | [0.93, 1] | 1.95 |
|  | AU26 | 622.54 | [0.85, 1] | 1.66 |
|  | AU43 | 438.83 | [0.72, 1] | 1.33 |
|  | AU53 | 755.07 | [0.94, 1] | 1.98 |

**Table S3**

*Raw Frequencies per Emotion*

| Emotion | AU1 | AU2 | AU6 | AU7 | AU9 | AU12 | AU14 | AU15 | AU17 | AU18 |
| --- | --- | --- | --- | --- | --- | --- | --- | --- | --- | --- |
| Admiration | 84 | 62 | 108 | 112 | 36 | 114 | 28 | 26 | 117 | 33 |
| Amae | 79 | 51 | 101 | 111 | 28 | 106 | 24 | 21 | 115 | 31 |
| Amusement | 80 | 63 | 145 | 124 | 48 | 144 | 32 | 36 | 113 | 36 |
| Awe | 86 | 61 | 109 | 118 | 47 | 120 | 26 | 34 | 113 | 39 |
| Being Moved | 72 | 49 | 111 | 112 | 30 | 118 | 17 | 18 | 109 | 21 |
| Determination | 79 | 64 | 99 | 115 | 28 | 105 | 20 | 26 | 120 | 34 |
| Elation | 79 | 56 | 128 | 121 | 36 | 134 | 24 | 28 | 115 | 29 |
| Excitement | 80 | 66 | 128 | 119 | 43 | 131 | 34 | 32 | 111 | 32 |
| Feeling Respected | 83 | 61 | 87 | 114 | 32 | 88 | 19 | 24 | 119 | 32 |
| Gratitude | 92 | 73 | 108 | 115 | 31 | 110 | 23 | 27 | 121 | 35 |
| Hope | 89 | 68 | 110 | 115 | 36 | 121 | 25 | 24 | 109 | 31 |
| Inspiration | 99 | 70 | 97 | 112 | 38 | 114 | 24 | 24 | 116 | 35 |
| Interest | 85 | 59 | 102 | 108 | 31 | 113 | 20 | 27 | 114 | 31 |
| Elevation | 93 | 59 | 94 | 111 | 30 | 97 | 24 | 28 | 125 | 35 |
| Positive Surprise | 87 | 75 | 125 | 109 | 35 | 128 | 26 | 26 | 111 | 28 |
| Pride | 79 | 66 | 108 | 114 | 30 | 120 | 20 | 22 | 121 | 28 |
| Relief | 88 | 67 | 115 | 118 | 34 | 115 | 25 | 25 | 120 | 34 |
| Schadenfreude | 67 | 49 | 121 | 107 | 26 | 126 | 25 | 22 | 106 | 25 |
| Sensory Pleasure | 85 | 66 | 129 | 130 | 44 | 135 | 35 | 40 | 116 | 44 |
| Sexual Desire | 79 | 56 | 115 | 112 | 36 | 112 | 22 | 23 | 106 | 32 |
| Tenderness | 75 | 53 | 131 | 116 | 40 | 130 | 25 | 28 | 106 | 36 |
| Triumph | 84 | 66 | 125 | 109 | 42 | 132 | 30 | 31 | 110 | 39 |
| Total | 1824 | 1360 | 2496 | 2522 | 781 | 2613 | 548 | 592 | 2513 | 720 |

**Table S4**

*Raw Frequencies per Emotion*

| Emotion | AU20 | AU23 | AU25 | AU26 | AU27 | AU43 | AU51 | AU52 | AU54 |
| --- | --- | --- | --- | --- | --- | --- | --- | --- | --- |
| Admiration | 60 | 26 | 157 | 146 | 30 | 124 | 131 | 141 | 51 |
| Amae | 48 | 21 | 151 | 138 | 25 | 116 | 135 | 145 | 46 |
| Amusement | 55 | 32 | 159 | 146 | 39 | 126 | 143 | 148 | 70 |
| Awe | 51 | 28 | 156 | 143 | 33 | 121 | 141 | 142 | 59 |
| Being Moved | 36 | 15 | 156 | 140 | 19 | 126 | 133 | 142 | 51 |
| Determination | 44 | 19 | 158 | 145 | 24 | 127 | 135 | 146 | 50 |
| Elation | 46 | 25 | 160 | 148 | 37 | 125 | 141 | 151 | 61 |
| Excitement | 57 | 32 | 159 | 147 | 43 | 117 | 142 | 150 | 47 |
| Feeling Respected | 47 | 21 | 158 | 143 | 23 | 122 | 134 | 141 | 44 |
| Gratitude | 46 | 24 | 158 | 144 | 29 | 127 | 140 | 149 | 48 |
| Hope | 44 | 23 | 155 | 147 | 28 | 126 | 137 | 147 | 52 |
| Inspiration | 54 | 25 | 153 | 143 | 29 | 121 | 140 | 149 | 50 |
| Interest | 42 | 21 | 156 | 143 | 28 | 127 | 136 | 155 | 46 |
| Elevation | 45 | 23 | 155 | 134 | 27 | 116 | 130 | 147 | 38 |
| Positive Surprise | 57 | 23 | 157 | 143 | 35 | 129 | 134 | 150 | 59 |
| Pride | 44 | 19 | 155 | 143 | 32 | 112 | 137 | 146 | 47 |
| Relief | 49 | 24 | 153 | 141 | 33 | 125 | 132 | 145 | 53 |
| Schadenfreude | 43 | 19 | 148 | 141 | 25 | 119 | 128 | 138 | 51 |
| Sensory Pleasure | 56 | 36 | 159 | 146 | 41 | 126 | 136 | 146 | 50 |
| Sexual Desire | 47 | 23 | 153 | 142 | 29 | 126 | 127 | 143 | 54 |
| Tenderness | 47 | 26 | 156 | 144 | 28 | 127 | 137 | 143 | 65 |
| Triumph | 52 | 30 | 157 | 146 | 38 | 127 | 134 | 145 | 47 |
| Total | 1070 | 535 | 3429 | 3153 | 675 | 2712 | 2983 | 3209 | 1139 |

**Table S5**

*Raw Frequencies (%) per Emotion*

| Emotion | AU1 | AU2 | AU6 | AU7 | AU9 | AU12 | AU14 | AU15 | AU17 | AU18 |
| --- | --- | --- | --- | --- | --- | --- | --- | --- | --- | --- |
| Admiration | 51.9 | 38.3 | 66.7 | 69.1 | 22.2 | 70.4 | 17.3 | 16 | 72.2 | 20.4 |
| Amae | 49.7 | 32.1 | 63.5 | 69.8 | 17.6 | 66.7 | 15.1 | 13.2 | 72.3 | 19.5 |
| Amusement | 49.4 | 38.9 | 89.5 | 76.5 | 29.6 | 88.9 | 19.8 | 22.2 | 69.8 | 22.2 |
| Awe | 53.8 | 38.1 | 68.1 | 73.8 | 29.4 | 75 | 16.2 | 21.2 | 70.6 | 24.4 |
| Being Moved | 44.7 | 30.4 | 68.9 | 69.6 | 18.6 | 73.3 | 10.6 | 11.2 | 67.7 | 13 |
| Determination | 48.8 | 39.5 | 61.1 | 71 | 17.3 | 64.8 | 12.3 | 16 | 74.1 | 21 |
| Elation | 48.8 | 34.6 | 79 | 74.7 | 22.2 | 82.7 | 14.8 | 17.3 | 71 | 17.9 |
| Excitement | 49.4 | 40.7 | 79 | 73.5 | 26.5 | 80.9 | 21 | 19.8 | 68.5 | 19.8 |
| Feeling Respected | 51.2 | 37.7 | 53.7 | 70.4 | 19.8 | 54.3 | 11.7 | 14.8 | 73.5 | 19.8 |
| Gratitude | 56.4 | 44.8 | 66.3 | 70.6 | 19 | 67.5 | 14.1 | 16.6 | 74.2 | 21.5 |
| Hope | 54.9 | 42 | 67.9 | 71 | 22.2 | 74.7 | 15.4 | 14.8 | 67.3 | 19.1 |
| Inspiration | 61.1 | 43.2 | 59.9 | 69.1 | 23.5 | 70.4 | 14.8 | 14.8 | 71.6 | 21.6 |
| Interest | 52.5 | 36.4 | 63 | 66.7 | 19.1 | 69.8 | 12.3 | 16.7 | 70.4 | 19.1 |
| Elevation | 58.1 | 36.9 | 58.8 | 69.4 | 18.8 | 60.6 | 15 | 17.5 | 78.1 | 21.9 |
| Positive Surprise | 54 | 46.6 | 77.6 | 67.7 | 21.7 | 79.5 | 16.1 | 16.1 | 68.9 | 17.4 |
| Pride | 49.1 | 41 | 67.1 | 70.8 | 18.6 | 74.5 | 12.4 | 13.7 | 75.2 | 17.4 |
| Relief | 55 | 41.9 | 71.9 | 73.8 | 21.2 | 71.9 | 15.6 | 15.6 | 75 | 21.2 |
| Schadenfreude | 43.5 | 31.8 | 78.6 | 69.5 | 16.9 | 81.8 | 16.2 | 14.3 | 68.8 | 16.2 |
| Sensory Pleasure | 52.5 | 40.7 | 79.6 | 80.2 | 27.2 | 83.3 | 21.6 | 24.7 | 71.6 | 27.2 |
| Sexual Desire | 50 | 35.4 | 72.8 | 70.9 | 22.8 | 70.9 | 13.9 | 14.6 | 67.1 | 20.3 |
| Tenderness | 46.3 | 32.7 | 80.9 | 71.6 | 24.7 | 80.2 | 15.4 | 17.3 | 65.4 | 22.2 |
| Triumph | 51.9 | 40.7 | 77.2 | 67.3 | 25.9 | 81.5 | 18.5 | 19.1 | 67.9 | 24.1 |
| Total | 51.5 | 38.4 | 70.5 | 71.2 | 22.1 | 73.8 | 15.5 | 16.7 | 71 | 20.3 |

**Table S6**

*Raw Frequencies (%) per Emotion*

| Emotion | AU20 | AU23 | AU25 | AU26 | AU27 | AU43 | AU51 | AU52 | AU54 |
| --- | --- | --- | --- | --- | --- | --- | --- | --- | --- |
| Admiration | 37 | 16 | 96.9 | 90.1 | 18.5 | 76.5 | 80.9 | 87 | 31.5 |
| Amae | 30.2 | 13.2 | 95 | 86.8 | 15.7 | 73 | 84.9 | 91.2 | 28.9 |
| Amusement | 34 | 19.8 | 98.1 | 90.1 | 24.1 | 77.8 | 88.3 | 91.4 | 43.2 |
| Awe | 31.9 | 17.5 | 97.5 | 89.4 | 20.6 | 75.6 | 88.1 | 88.8 | 36.9 |
| Being Moved | 22.4 | 9.3 | 96.9 | 87 | 11.8 | 78.3 | 82.6 | 88.2 | 31.7 |
| Determination | 27.2 | 11.7 | 97.5 | 89.5 | 14.8 | 78.4 | 83.3 | 90.1 | 30.9 |
| Elation | 28.4 | 15.4 | 98.8 | 91.4 | 22.8 | 77.2 | 87 | 93.2 | 37.7 |
| Excitement | 35.2 | 19.8 | 98.1 | 90.7 | 26.5 | 72.2 | 87.7 | 92.6 | 29 |
| Feeling Respected | 29 | 13 | 97.5 | 88.3 | 14.2 | 75.3 | 82.7 | 87 | 27.2 |
| Gratitude | 28.2 | 14.7 | 96.9 | 88.3 | 17.8 | 77.9 | 85.9 | 91.4 | 29.4 |
| Hope | 27.2 | 14.2 | 95.7 | 90.7 | 17.3 | 77.8 | 84.6 | 90.7 | 32.1 |
| Inspiration | 33.3 | 15.4 | 94.4 | 88.3 | 17.9 | 74.7 | 86.4 | 92 | 30.9 |
| Interest | 25.9 | 13 | 96.3 | 88.3 | 17.3 | 78.4 | 84 | 95.7 | 28.4 |
| Elevation | 28.1 | 14.4 | 96.9 | 83.8 | 16.9 | 72.5 | 81.2 | 91.9 | 23.8 |
| Positive Surprise | 35.4 | 14.3 | 97.5 | 88.8 | 21.7 | 80.1 | 83.2 | 93.2 | 36.6 |
| Pride | 27.3 | 11.8 | 96.3 | 88.8 | 19.9 | 69.6 | 85.1 | 90.7 | 29.2 |
| Relief | 30.6 | 15 | 95.6 | 88.1 | 20.6 | 78.1 | 82.5 | 90.6 | 33.1 |
| Schadenfreude | 27.9 | 12.3 | 96.1 | 91.6 | 16.2 | 77.3 | 83.1 | 89.6 | 33.1 |
| Sensory Pleasure | 34.6 | 22.2 | 98.1 | 90.1 | 25.3 | 77.8 | 84 | 90.1 | 30.9 |
| Sexual Desire | 29.7 | 14.6 | 96.8 | 89.9 | 18.4 | 79.7 | 80.4 | 90.5 | 34.2 |
| Tenderness | 29 | 16 | 96.3 | 88.9 | 17.3 | 78.4 | 84.6 | 88.3 | 40.1 |
| Triumph | 32.1 | 18.5 | 96.9 | 90.1 | 23.5 | 78.4 | 82.7 | 89.5 | 29 |
| Total | 30.2 | 15.1 | 96.8 | 89 | 19.1 | 76.6 | 84.2 | 90.6 | 32.2 |

**Table S7**

*Summary of Findings for Hypothesis Tests Across Analyses*

| Emotion | Nonparametric χ*^2^* tests | | | Network Analyses | | |  |
| --- | --- | --- | --- | --- | --- | --- | --- |
|  | *Hyp* - **Sig** | *Hyp -*  Non-Sig | Not Hyp - **Sig** | *Hyp* - **Sig** | *Hyp -*  Non-Sig | Not Hyp - **Sig** |  |
| Admiration | ***12**** | *---* | **1^*^, 2^^^, 6^*^, 7^*^, 17^*^, 25^+^, 26^+^, 43^+^, 52^+^** | *---* | *12** | *---* |  |
| Amae | ***6*, 12**** | *---* | **1^^^, 2^^^, 7^*^, 17^*^, 25^+^, 26^+^, 43^*^, 52^+^** | *---* | *6*, 12** | *---* |  |
| Amusement | ***6^+,^ 7^+,^ 12^+,^ 25^+,^ 26^+^*** | *---* | **1^^^, 2^^^, 17^*^, 43^+^, 52^+^** | ***6^+^*, *7^+^*, *12^+^*** | *25****^+^****, 26****^+^*** | *---* |  |
| Awe | ***1*, 25^+^, 26^+^*** | *---* | **2^^^, 6^*^, 7^*^, 12^+^, 17^*^, 43^+^, 52^+^** | *---* | *1*, 25****^+^****, 26****^+^*** | *---* |  |
| Being Moved | ***6*, 7*, 12*, 43^+^*** | *---* | **1^^^, 2^^^, 12^*^, 17^*^, 25^+^, 26^+^, 52^+^** | *---* | *6*, 7*, 12*, 43****^+^*** | *---* |  |
| Determination | ***1^, 7**** | *---* | **2^^^, 6^*^, 12^*^, 17^*^, 25^+^, 26^+^, 43^+^, 52^+^** | *---* | *1^, 7** | **17*** |  |
| Elation | ***1^, 2^, 6^+^, 12^+^, 17*, 25^+^, 26^+^*** | *---* | **7^*^, 43^+^, 52^+^** | ***6^+^*, *12^+^*, *25^+^*, *26^+^*** | *1^, 2^, 17** | **7*, 52^+^** |  |
| Excitement | ***1^, 2^, 6^+^, 12^+^, 25^+^, 26^+^*** | *---* | **7^*^, 17^*^, 43^*^, 52^+^** | ***6^+^*, *12^+^*** | *1^, 2^, 26****^+^*** | **52*^+^*** |  |
| Feeling Respected | ***6*, 7*, 12**** | *---* | **1^*^, 2^^^, 17^*^, 25^+^, 26^+^, 43^+^, 52^+^** | *---* | *6*, 7*, 12** | *---* |  |
| Gratitude | ***12**** | *---* | **1^*^, 2^^^, 6^*^, 7^*^, 17^*^, 25^+^, 26^+^, 43^+^, 52^+^** | *---* | *12** | **1*, 2^, 17*** |  |
| Hope | ***1*, 2^, 6*, 12*, 17**** | *---* | **7^*^, 25^+^, 26^+^, 43^+^, 52^+^** | ***1*, 2^*** | *6*, 12*, 17** | *---* |  |
| Inspiration | ***1*, 12*, 25^+^*** | *---* | **2^^^, 6^*^, 7^*^, 17^*^, 26^+^, 43^+^, 52^+^** | ***1**** | *12*, 25^+^* | **2^** |  |
| Interest | ***1**** | *---* | **2^, 6*, 7*, 12*, 17*, 25+, 26+, 43+, 52^+^** | ***---*** | *1** | **52^+^** |  |
| Moral Elevation | ***6*, 12*, 25^+^, 26^+^*** | *---* | **1*, 2^, 7*, 17+, 43*, 52^+^** | ***---*** | *6*, 12*, 25^+^, 26^+^* | **1*, 17^+^, 52^+^** |  |
| Positive Surprise | ***1*, 2^, 6^+^, 12^+^, 25^+^, 26^+^*** | *---* | **7*, 17*, 43+, 52^+^** | ***2^, 6^+^, 12^+^*** | *1*, 25****^+^****, 26****^+^*** | **43^+^, 52^+^** |  |
| Pride | ***7*, 12**** | *---* | **1^, 2^, 6*, 17+, 25+, 26+, 43*, 52^+^** | ***---*** | *7*, 12** | **2^, 17^+^** |  |
| Relief | ***12*, 25^+^, 26^+^, 43^+^*** | *---* | **1*, 2^, 6*, 7*, 17+, 52^+^** | ***---*** | *12*, 25****^+^****, 26****^+^****, 43****^+^*** | **1*, 2^, 17^+^** |  |
| Schadenfreude | ***6^+^, 12^+^*** | *---* | **1^, 2^, 7*, 17*, 25+, 26+, 43+, 52^+^** | ***6^+^, 12^+^*** | *---* | **26^+^** |  |
| Sensory Pleasure | ***6^+^, 7^+^, 12^+^, 25^+^, 26^+^, 43^+^*** | *---* | **1*, 2^, 17*, 52^+^** | ***6^+^, 7^+^, 12^+^*** | *25****^+^****, 26****^+^****, 43****^+^*** | **---** |  |
| Sexual Desire | ***6*, 7*, 12*, 25^+^*** | *---* | **1*, 2^, 17*, 26+, 43+, 52^+^** | ***6**** | *7*, 12*, 25****^+^*** | **43^+^** |  |
| Tenderness | ***6^+^, 12^+^*** | *---* | **1^, 2^, 7*, 17*, 25+, 26+, 43+, 52^+^** | ***6^+^, 12^+^*** | *---* | **---** |  |
| Triumph | ***6^+^, 7*, 12^+^, 25^+^, 26^+^*** | *---* | **1*, 2^, 17*, 43+, 52^+^** | ***6^+^, 12^+^*** | *7*, 25****^+^****, 26****^+^*** | **---** |  |

*Note*. Columns names respectively refer to *Hypothesized* and **Significant**, *Hypothesized* but Non-Significant, Not Hypothesized but **Significant**. Numbers indicate each Action Unit (AU). Predictions italicized, significant results in bold, ^#^ = frequencies less than 25%, ^ = frequencies greater than 25%, * = frequencies greater than 50%, ^+^ = frequencies greater than 75%.

**References**

Algoe, S. B., & Zhaoyang, R. (2016). Positive psychology in context: Effects of expressing gratitude in ongoing relationships depend on perceptions of enactor responsiveness. *The Journal of Positive Psychology*, *11*(4), 399-415.

Ambadar, Z., Cohn, J. F., & Reed, L. I. (2009). All smiles are not created equal: Morphology and timing of smiles perceived as amused, polite, and embarrassed/nervous. *Journal of Nonverbal Behavior*, *33*(1), 17-34.

Aron, A., Aron, E. N., & Smollan, D. (1992). Inclusion of other in the self scale and the structure of interpersonal closeness. *Journal of Personality and Social Psychology*, *63*(4), 596-612.

Bänziger, T., Mortillaro, M., & Scherer, K. R. (2012). Introducing the Geneva Multimodal expression corpus for experimental research on emotion perception. *Emotion*, *12*(5), 1161-1179.

Boecker, L., Likowski, K. U., Pauli, P., & Weyers, P. (2015). The face of schadenfreude: Differentiation of joy and schadenfreude by electromyography. *Cognition & Emotion*, *29*(6), 1117-1125.

Campos, B., Shiota, M. N., Keltner, D., Gonzaga, G. C., & Goetz, J. L. (2013). What is shared, what is different? Core relational themes and expressive displays of eight positive emotions. *Cognition & Emotion*, *27*(1), 37-52.

Cicourel, A. V. (1996). Ecological validity and'white room effects': The interaction of cognitive and cultural models in the pragmatic analysis of elicited narratives from children. *Pragmatics & Cognition*, *4*(2), 221-264.

Clauset, A., Newman, M., & Moore, C. (2004). Finding community structure in very large networks. Physical Review E - Statistical Physics, Plasmas, Fluids, and Related Interdisciplinary Topics, 70(6), 6. https://doi.org/10.1103/PhysRevE.70.066111

Cohen-Chen, S., van Kleef, G. A., Crisp, R. J., & Halperin, E. (2019). Dealing in hope: Does observing hope expressions increase conciliatory attitudes in intergroup conflict?. *Journal of Experimental Social Psychology*, *83*, 102-111.

Cordaro, D. T., Sun, R., Keltner, D., Kamble, S., Huddar, N., & McNeil, G. (2018). Universals and cultural variations in 22 emotional expressions across five cultures. *Emotion*, *18*(1), 75-93.

Dael, N., Mortillaro, M., & Scherer, K. R. (2012). Emotion expression in body action and posture. *Emotion*, *12*(5), 1085-1101.

Dupré, D., Krumhuber, E. G., Küster, D., & McKeown, G. J. (2020). A performance comparison of eight commercially available automatic classifiers for facial affect recognition. *PloS one*, *15*(4), e0231968.

Ekman, P. (1982). Methods for measuring facial action. In K.R. Scherer & P. Ekman (Eds.), *Handbook of methods in nonverbal behavior research* (pp. 45–90). Cambridge: Cambridge University.

Elfenbein, H. A., Beaupré, M., Lévesque, M., & Hess, U. (2007). Toward a dialect theory: Cultural differences in the expression and recognition of posed facial expressions. *Emotion*, 7, 131–146. http://dx.doi.org/10.1037/1528-3542.7.1.131

Fernández-Dols, J. M., Carrera, P., & Crivelli, C. (2011). Facial behavior while experiencing sexual excitement. *Journal of Nonverbal Behavior*, *35*(1), 63-71.

Gonzaga, G. C., Keltner, D., Londahl, E. A., & Smith, M. D. (2001). Love and the commitment problem in romantic relations and friendship. *Journal of Personality and Social Psychology*, *81*(2), 247-262.

Gonzaga, G. C., Turner, R. A., Keltner, D., Campos, B., & Altemus, M. (2006). Romantic love and sexual desire in close relationships. *Emotion*, *6*(2), 163-179.

Hwang, H. C., & Matsumoto, D. (2014). Cultural differences in victory signals of triumph. *Cross-Cultural Research*, *48*(2), 177-191.

Johnson, K. J., & Fredrickson, B. L. (2005). “We all look the same to me” Positive emotions eliminate the own-race bias in face recognition. *Psychological Science*, *16*(11), 875-881.

Kalokerinos, E. K., Greenaway, K. H., Pedder, D. J., & Margetts, E. A. (2014). Don’t grin when you win: The social costs of positive emotion expression in performance situations. *Emotion*, *14*(1), 180-186.

Keltner, D., & Haidt, J. (1999). Social Functions of Emotions at Four Levels of Analysis. *Cognition and Emotion*, *13*(5), 505–521. https://doi.org/10.1080/026999399379168

Kitayama, S., Mesquita, B., & Karasawa, M. (2006). Cultural affordances and emotional experience: socially engaging and disengaging emotions in Japan and the United States. *Journal of Personality and Social Psychology*, *91*(5), 890-903.

Krumhuber, E. G., & Scherer, K. R. (2011). Affect bursts: dynamic patterns of facial expression. *Emotion*, *11*(4), 825-841.

Larsen, J. T., & McGraw, A. P. (2014). The case for mixed emotions. *Social and Personality Psychology Compass*, *8*(6), 263-274.

Lazarus, R. S. (1999). Hope: An emotion and a vital coping resource against despair. *Social Research*, 653-678.

Li, J., & Fischer, K. W. (2007). Respect as a positive self-conscious emotion in European Americans and Chinese. *The self-conscious emotions: Theory and research*, 224-242.

Lyng, S. (2018). Excitement: Risk and authentic emotion. In *Emotions, Everyday Life and Sociology* (pp. 88-103). Routledge.

Madore, K. P., Jing, H. G., & Schacter, D. L. (2019). Selective effects of specificity inductions on episodic details: evidence for an event construction account. *Memory*, *27*(2), 250-260.

Mielke, A., Waller, B. M., Pérez, C., Rincon, A. V., Duboscq, J., & Micheletta, J. (2022). NetFACS: Using network science to understand facial communication systems. *Behavior Research Methods*, *54*(4), 1912-1927.

Monroy, M., & Keltner, D. (2022). Awe as a pathway to mental and physical health. *Perspectives on Psychological Science*, 17456916221094856.

Mortillaro, M., Mehu, M., & Scherer, K. R. (2011). Subtly different positive emotions can be distinguished by their facial expressions. *Social Psychological and Personality Science*, *2*(3), 262-271.

Newman, M. (2010). *Networks: An Introduction*. Oxford University Press. https://doi.org/10.1093/acprof:oso/9780199206650.001.0001

Niiya, Y., Ellsworth, P. C., & Yamaguchi, S. (2006). Amae in Japan and the United States: An exploration of a" culturally unique" emotion. *Emotion*, *6*(2), 279-295.

Onu, D., Kessler, T., & Smith, J. R. (2016). Admiration: A conceptual review. *Emotion Review*, *8*(3), 218-230.

Piepiora, P. (2021). Assessment of personality traits influencing the performance of men in team sports in terms of the big five. *Frontiers in Psychology*, 1901.

Reeve, J. (1993). The face of interest. *Motivation & Emotion*, *17*(4), 353-375.

Ricci-Bitti, P., Caterina, R., & Garotti, P. (1996). Different behavioral markers in different smiles. In N. H. Frijda (Ed.), *Proceedings of the eighth Conference of the International Society for Research on Emotions* (pp. 297–301). Storrs, CT: ISRE.

Rozin, P., & Cohen, A. B. (2003). High frequency of facial expressions corresponding to confusion, concentration, and worry in an analysis of naturally occurring facial expressions of Americans. *Emotion*, *3*(1), 68-75.

Rychlowska, M., Jack, R. E., Garrod, O. G., Schyns, P. G., Martin, J. D., & Niedenthal, P. M. (2017). Functional smiles: Tools for love, sympathy, and war. *Psychological Science*, *28*(9), 1259-1270.

Schnall, S., & Roper, J. (2012). Elevation puts moral values into action. *Social Psychological and Personality Science*, *3*(3), 373-378.

Schubert, T. W., Zickfeld, J. H., Seibt, B., & Fiske, A. P. (2018). Moment-to-moment changes in feeling moved match changes in closeness, tears, goosebumps, and warmth: Time series analyses. *Cognition & Emotion*, *32*(1), 174-184.

Shiota, M. N., Campos, B., & Keltner, D. (2003). The faces of positive emotion: Prototype displays of awe, amusement, and pride. *Annals of the New York Academy of Sciences*, *1000*(1), 296-299.

Silvia, P. J. (2008). Interest—The curious emotion. *Current Directions in Psychological Science*, *17*(1), 57-60.

Stratton, G. M. (1928). The function of emotion as shown particularly in excitement. *Psychological Review*, *35*(5), 351-366.

Sun, R., Hou, W. K., Hui, B. P., Siu, N. Y. F., Engels, T., & Sauter, D. A. (2021). Perception and evaluation of 23 positive emotions in Hong Kong and the Netherlands. *Frontiers in Psychology*, *12*, 579474.

Thomson, A. L., & Siegel, J. T. (2017). Elevation: A review of scholarship on a moral and other-praising emotion. *The Journal of Positive Psychology*, *12*(6), 628-638.

Thrash, T. M., & Elliot, A. J. (2003). Inspiration as a psychological construct. *Journal of Personality and Social Psychology*, *84*(4), 871-889.

Thrash, T. M., Moldovan, E. G., Oleynick, V. C., & Maruskin, L. A. (2014). The psychology of inspiration. *Social and Personality Psychology Compass*, *8*(9), 495-510.

Tracy, J. L., & Matsumoto, D. (2008). The spontaneous expression of pride and shame: Evidence for biologically innate nonverbal displays. *Proceedings of the National Academy of Sciences*, *105*(33), 11655-11660.

Tracy, J. L., & Robins, R. W. (2004). Show your pride: Evidence for a discrete emotion expression. *Psychological Science*, *15*(3), 194-197.

Tsai, J. L., Knutson, B., & Fung, H. H. (2006). Cultural variation in affect valuation. *Journal of Personality and Social Psychology*, *90*(2), 288-307.

Vrticka, P., Lordier, L., Bediou, B., & Sander, D. (2014). Human amygdala response to dynamic facial expressions of positive and negative surprise. *Emotion*, *14*(1), 161-169.

Wallbott, H. G. (1998). Decoding emotions from facial expression: Recent developments and findings. *European Review of Social Psychology*, *9*(1), 191-232.

Zickfeld, J. H., Schubert, T. W., Seibt, B., Blomster, J. K., Arriaga, P., Basabe, N., ... & Fiske, A. P. (2019). Kama muta: Conceptualizing and measuring the experience often labelled being moved across 19 nations and 15 languages. *Emotion*, *19*(3), 402-424.
